# Breaking of the Up‐Down Symmetry of DNA Origami on a Solid Substrate

**DOI:** 10.1002/anie.202507613

**Published:** 2025-10-12

**Authors:** Gangamallaiah Velpula, Emilia Tomm, Boxuan Shen, Kunal S. Mali, Adrian Keller, Steven De Feyter

**Affiliations:** ^1^ Department of Chemistry Division of Molecular Imaging and Photonics KU Leuven Leuven B‐3001 Belgium; ^2^ Paderborn University Technical and Macromolecular Chemistry Warburger Str.100 33098 Paderborn Germany; ^3^ Department of Medical Biochemistry and Biophysics Karolinska Institutet Stockholm 17177 Sweden

**Keywords:** Atomic force microscopy, Chirality, DNA origami, oxDNA simulations

## Abstract

Controlling the surface orientation of DNA origami nanostructures (DON) is crucial for applications in nanotechnology and materials science. While previous work utilized various DON modifications, simple methods for controlling their landing orientation remain scarce. Here, we demonstrate a straightforward approach to control the adsorption orientation of chiral double‐L (CDL) DON on mica by tuning magnesium ion (Mg^2^⁺) concentration and exploiting global shape distortions. Using atomic force microscopy (AFM), we analyzed the resulting distribution of the mirror‐image orientations, referred to as S and Z orientations, at both buffer/mica and air/mica interfaces and identified conditions resulting in homogenous CDL orientation of 100% S. These results demonstrate how DON conformation and ionic environments influence DON orientation, offering insights for precise nanostructure deposition.

## Graphical abstract

In this study, the surface orientation of chiral double‐L (CDL) DNA origami on mica was controlled by tuning magnesium ion (Mg^2^⁺) concentration. This simple yet powerful method, exploiting global shape distortions, achieved 100% S orientation enabling precise nanostructure deposition.

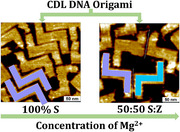

Because of their nanoscale precision, design versatility, and self‐assembly capabilities DNA origami nanostructures (DON) have emerged as powerful templates for fabricating complex structures.^[^ ^1^, ^2^, ^3^ ^]^ Defined control over the surface orientation of DON is paramount for realizing their potential in diverse applications, across metamaterials,^[^ ^1^, ^4^ ^]^ and chiral sensing^[^ ^5^, ^6^ ^]^ to data storage^[^ ^7^, ^8^ ^]^ and nanoelectronics.^[^ ^9^, ^10^ ^]^ For instance, the performance of chiral metamaterials depends on the consistent orientation of their constituent chiral DON.^[^ ^1^ ^]^ Random surface adsorption leads to averaging effects, significantly decreasing or even cancelling the desired chiroptical response.^[^ ^1^, ^11^ ^]^ However, achieving controlled landing orientation of DON on surface remains a critical challenge.^[^ ^12^ ^]^ Therefore, developing robust strategies to control DON surface orientation is essential for maximizing their utility in applications.

Early studies on DON focused on understanding basic principles and developing methods to construct complex nanostructures.^[^ ^13^, ^14^ ^]^ The deposition of DON on solid substrates has been extensively studied on hydrophilic substrates such as mica^[^ ^13^, ^15^, ^16^, ^17^ ^]^ and SiO_2_.^[^ ^18^, ^19^, ^20^ ^]^ Recent efforts have also focused on adsorbing DON on conductive surfaces such as TiO_2_,^[^ ^21^ ^]^ graphene,^[^ ^22^ ^]^ and highly oriented pyrolytic graphite^[^ ^23^, ^24^ ^]^ to expand its applications in various fields including biosensors^[^ ^25^ ^]^ and catalysis.^[^ ^26^ ^]^

There are few studies exploring DON adsorption with a specified orientation. By incorporating protruding staple strands into the DON design, it is possible to control their preferred orientations on the surface.^[^ ^1^, ^12^ ^]^ Noncovalent binding of DNA intercalators, which insert between base pairs and cause the unwinding of the DNA double helix, has also been employed. The unwinding effect modulates the helicity mismatch in DON, significantly influencing the internal stress and global conformation of the structure.^[^ ^27^ ^]^ However, these approaches often require the introduction of modifications either by replacing a large number of staple strands or by loading with other potentially toxic molecules to control orientation, which limits their scalability and versatility. Thus, developing a more straightforward, fundamental method to control DON orientation on surfaces is of great significance.

Here, we present a simple approach to control the DON orientation upon deposition on mica, an intrinsically negatively charged substrate. By varying the Mg^2^⁺ concentration of the buffer solution, we demonstrate the ability to control the orientation of a chiral DON shape on the mica surface (Figure 1). Immobilization of DNA on mica typically requires divalent cations, such as Mg^2^⁺, to facilitate the formation of salt bridges between the DNA back‐bone and the mica surface.^[^ ^17^ ^]^ A chiral double‐L (**CDL**) DON was used that can adopt either an **S** or **Z** orientation upon adsorption.

**Figure 1:**
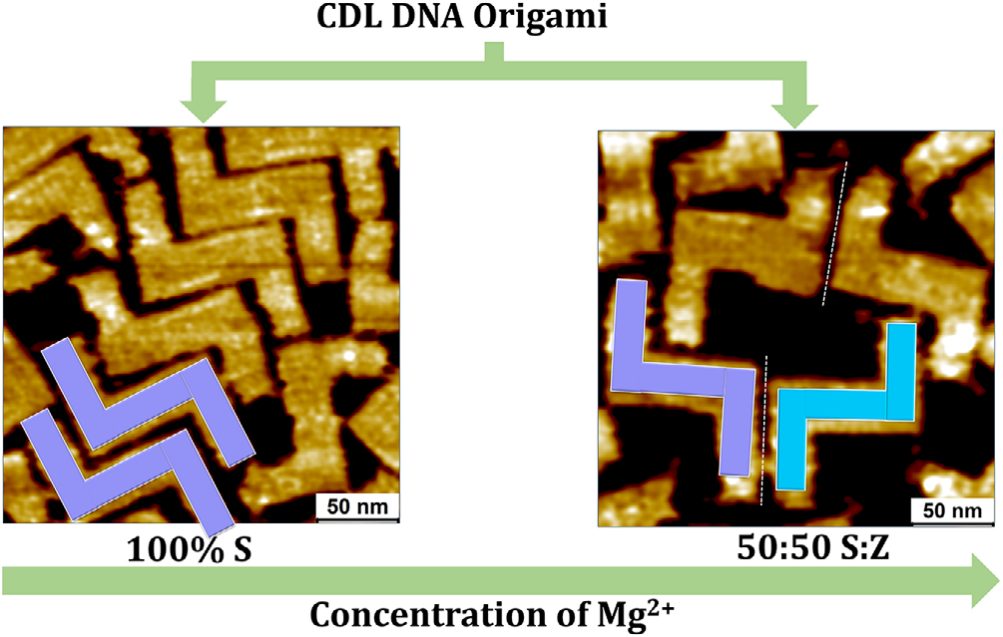
Schematic representations and high‐resolution AFM images (scale bar is 50 nm) illustrating the mirror‐image **S** and **Z** orientations of **CDL** DON adsorbed on mica as a function of Mg^2^⁺ concentration. A white dashed line separates the two **CDL** DON orientations (right).

While adsorption orientation of the **CDL** DON has previously been controlled by protruding staple strand extensions.^[^ ^1^ ^]^ Here we demonstrate that adsorption can also be biased by global shape distortions as revealed by oxDNA simulations. **CDL** DON adsorption on mica was probed by atomic force microscopy (AFM), both for dry samples as well as at the liquid‐solid interface. Distributions of **S** and **Z** orientations are shown to depend dramatically on the Mg^2+^ concentration, ranging from randomly oriented **CDLs** to exclusive **S**. The results are explained by considering Mg^2^⁺ induced conformational transitions in the 3D shape of the **CDL** DON. At low Mg^2+^ concentrations, both of its arms are partially rolled up on one of its faces, so that adsorption with this face toward the surface is suppressed.

A 1x TAE buffer solution (40 µL) of **CDL** DON containing different concentrations of Mg^2^⁺ ions (3.5–100 mM) was deposited onto mica and subsequently imaged using AFM at the TAE buffer/mica interface. The **S** and **Z** DON orientations are clearly distinguishable (Figure 2). At low Mg^2+^ concentration (≤5.0 mM of Mg^2+^), the surface is almost exclusively populated by **S** orientations (Figure 3a). As the Mg^2^⁺ concentration increases, the fraction of **Z** orientations increases, reaching an equal distribution of both DON orientations at 100 mM Mg^2^⁺.

**Figure 2:**
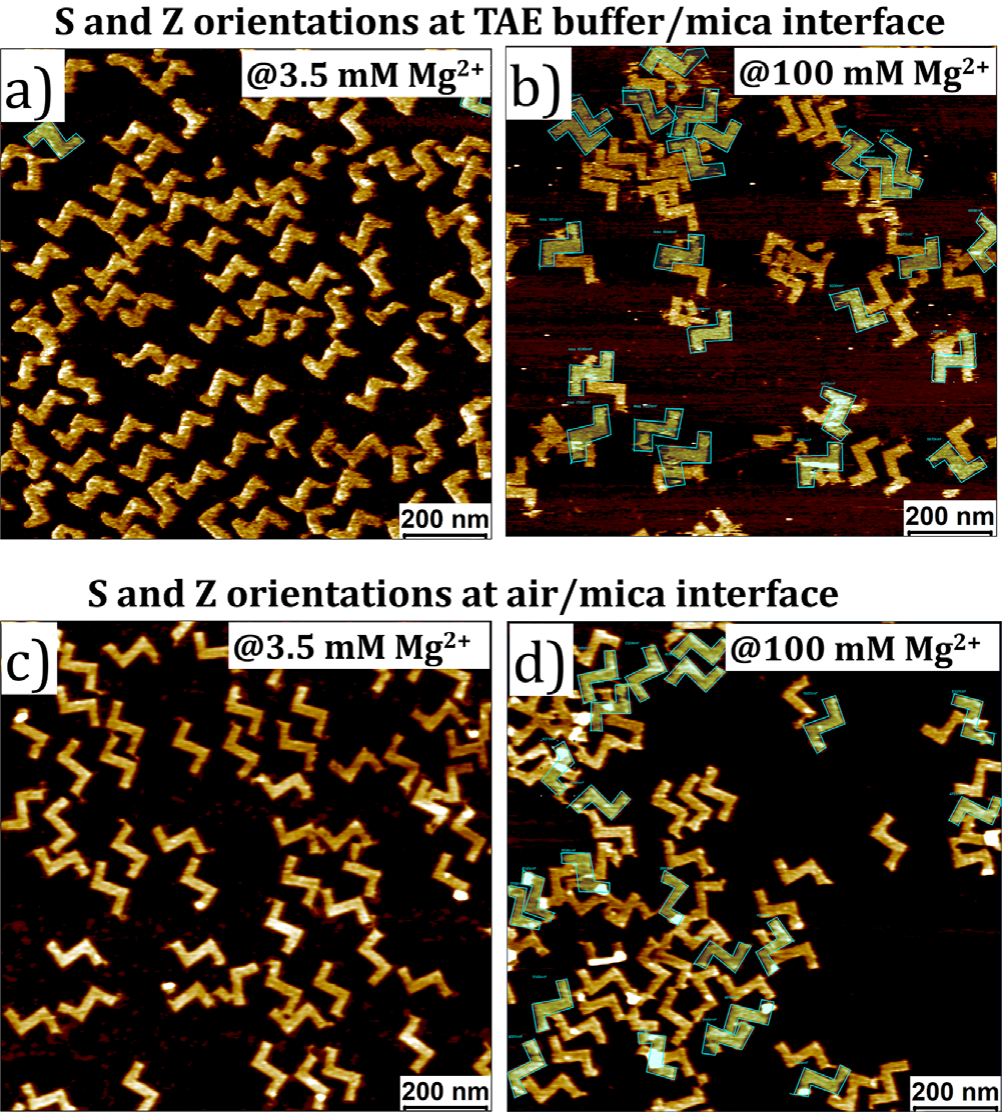
a, b) Large‐scale AFM images (scale bar is 200 nm) of **CDL** DON at the 1x TAE buffer/mica interface containing a) 3.5 mM Mg^2+^ and b) 100 mM Mg^2+^ (top). (c, d) Large‐scale AFM images (scale bar is 200 nm) of **CDL** DON at the air/mica interface containing c) 3.5 mM Mg^2+^ and d) 100 mM Mg^2+^ (bottom). **Z** orientations of **CDL** DON are highlighted in cyan. AFM images showing concentration dependent chiral induction at the 1x TAE buffer/mica and air/mica interfaces are provided in Figure S1–S16.

**Figure 3:**
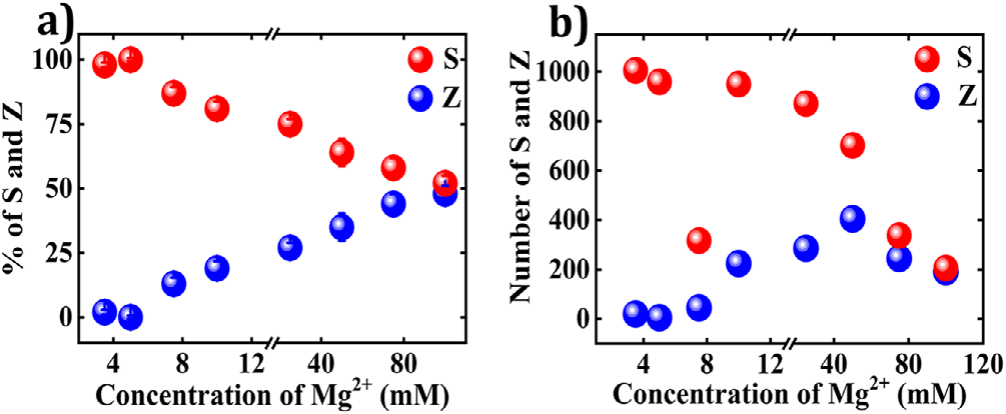
**S** and **Z** orientations of the **CDL** DON as a function of Mg^2+^ concentration at TAE buffer/mica interface. The population distribution of **S** and **Z** orientations is analyzed as a function of Mg^2+^ concentration expressed in a) percentage and b) absolute numbers. Each data point is based on several hundred to a thousand DON, obtained from six different images. Refer to the Supporting Information for AFM images (Figures S1–S8) and data analysis (Tables S1–S8). [**CDL**] = 0.55 nM. For [Mg^2+^] ≤5 mM, [**CDL**] = 1.1 nM as at lower **CDL** concentrations no DON adsorption was observed.

A detailed analysis of individual DON counts versus Mg^2+^ concentration revealed that this trend is mainly caused by the drastic decrease in the **S** orientations upon increasing Mg^2+^ concentration, while **Z** orientations exhibited an initial increase (until 50 mM Mg^2+^) followed by a decrease at higher concentrations (Figure 3b).

In general, it is expected that higher Mg^2+^ concentrations would increase the attractive interactions between the DON and the mica surface, leading to a higher surface coverage. However, our AFM images show the opposite effect. To understand this, we determined the total number of **CDL** DON (**S + Z**) as a function of Mg^2+^ concentration (Figure S20). The average surface coverage of the DON gradually decreases as the Mg^2+^ concentration increases. This counterintuitive result is supported by the visible aggregation of DON at 100 mM Mg^2+^ (Figure 2b–d), which indicates local charge inversion of the DON surface due to the high‐density binding of Mg^2+^ ions. This overcompensation of charge leads to aggregation in solution, thereby reducing the number of individual DON available to adsorb onto the mica interface.

Subsequently, a 1x TAE buffer solution (10 µL) of **CDL** DON containing variable concentrations of Mg^2^⁺ ions (3.5–100 mM) was deposited onto mica. After 1 min, the droplet was removed and the surface was washed with water (high purity ICP‐MS grade, pico‐pure plus). The mica surface was then dried using compressed air and characterized using AFM at the air/mica interface.

Again, this process predominantly yielded a surface populated by **CDL** shapes in **S** orientations at a Mg^2^⁺ concentration of 3.5 mM (Figure 4a). Upon increasing the Mg^2+^ concentration, the percentage of **S** orientations decreased, reaching a plateau (∼70%) at 50 mM. A condition of equal surface coverage of **S** and **Z** orientations was not reached.

**Figure 4:**
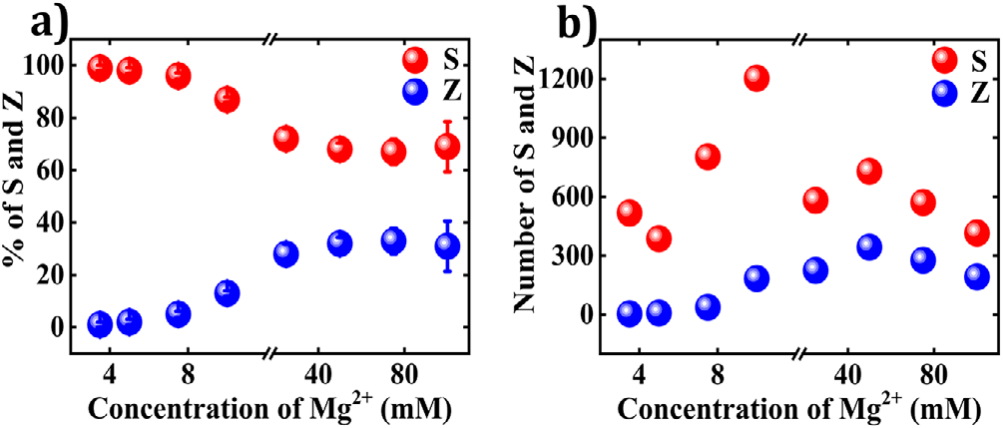
**S** and **Z** orientations of the **CDL** DON as a function of Mg^2+^ concentration at the air/mica interface. The population distribution of **S** and **Z** orientations is analyzed as a function of Mg^2+^ concentration expressed in a) percentage and b) absolute numbers. Each data point is based on several hundred to a thousand DON, obtained from six different images. Refer to the Supporting Information for AFM images (Figures S9–S16) and data analysis (Tables S9–S16). [**CDL**] = 2.75 nM. For [Mg^2+^] ≤5 mM, [**CDL**] = 5.5 nM as at lower CDL concentrations no DON was observed.

The air/mica interface showed a more complex trend than the buffer/mica interface. In both cases, both **S** and **Z** orientations of DON increased with Mg^2^⁺ concentration but peaked at different concentrations (10 mM for **S**, 50 mM for **Z**), before decreasing at higher concentrations.

Comparing both interfaces, the buffer/mica interface showed a more pronounced shift towards a 50:50 ratio. Comparing the absolute numbers of both conformations in Figures 3b and 4b, it is obvious that there are fewer **S** orientations in the dry state at low Mg^2+^. Two factors may account for these differences. First, longer incubation times during the liquid AFM measurements may give the DON more time to settle on the surface and adopt their preferred orientation. Second, washing may remove some of the adsorbed DON.

To gain a deeper understanding of the surface density variations of **S** and **Z** conformations at the air/mica and buffer/mica interfaces, we conducted a control experiment. Initially, AFM measurements were performed at the buffer/mica interface with a high concentration (100 mM) of Mg^2^⁺, revealing a 51:49 **S:Z** surface density ratio (Figure S17 and Table S17). Subsequently, the droplet was removed from the mica surface and dried using compressed air followed by AFM characterization of the dry surface. Interestingly, this yielded 61:39 **S:Z** ratio (Figure S18 and Table S18). The variation in surface density between the air/mica and buffer/mica interfaces was slightly smaller in this control experiment compared to the one, where the DON solution droplet was only kept for one minute (Figure 4a). In contrast, the droplet in the control experiment was left for over an hour. This suggests that while both factors contribute to the observed differences, **Z**‐shaped DON desorption during washing appears to be the dominant factor responsible for the unequal distribution of **S** and **Z** orientations at high Mg^2+^ concentrations.

It was previously shown that the orientation of adsorbed **CDL** DON can be controlled by extending the staples of its faces with single stranded overhangs that act as entropic springs and hinder **CDL** DON adsorption with this face toward the surface.^[^ ^1^, ^12^ ^]^ In the current experiments, the same was achieved without staple overhangs at low Mg^2+^ concentrations. Therefore, we hypothesize that adsorption of the **CDL** DON may be biased by global shape distortions that act in a similar manner. Indeed, the oxDNA simulations shown in Figure 5a reveal that both arms of the CDL are partially rolled up on the same face. This hinders adsorption with this face pointing down toward the mica surface, i.e., in **Z** orientation. This observation aligns with the AFM investigations. See Supporting Information for the design aspects of **CDL** DON and additional oxDNA simulations addressing the origin of its arms’ curvature. When the Mg^2^⁺ concentration is lowered, the arms of the DON become less sharply defined and may exhibit streaky features due to increased dynamic movement (Figure S1, S2, S19). Therefore, this global shape distortion explains why at low Mg^2+^ concentrations, virtually all **CDL** DON adsorb in **S** orientation, with the rolled‐up arms pointing away from the surface (Figure 5a). To understand how salt concentration influences the curvature of the **CDL** DON arms, we performed oxDNA simulations as a function of ionic strength. Since oxDNA is not able to assess the specific effects of Mg^2+^ binding^[^ ^28^ ^]^ we settled on different Na⁺ concentrations (0.5 M, 2.0 M, and 5.0 M). The results in Figure 5 indicate that the curvature of the **CDL** DON arm gradually decreases as the Na⁺ concentration increases (see also Figure S22 and Table S19). However, even the highest concentration of Na⁺ (5.0 M) used in the simulations does not completely eliminate the arms’ curvature. Given that Na⁺ and Mg^2^⁺ interact with DNA differently, however, Mg^2^⁺ may have an even stronger influence on reducing the curvature of the arm.

**Figure 5:**
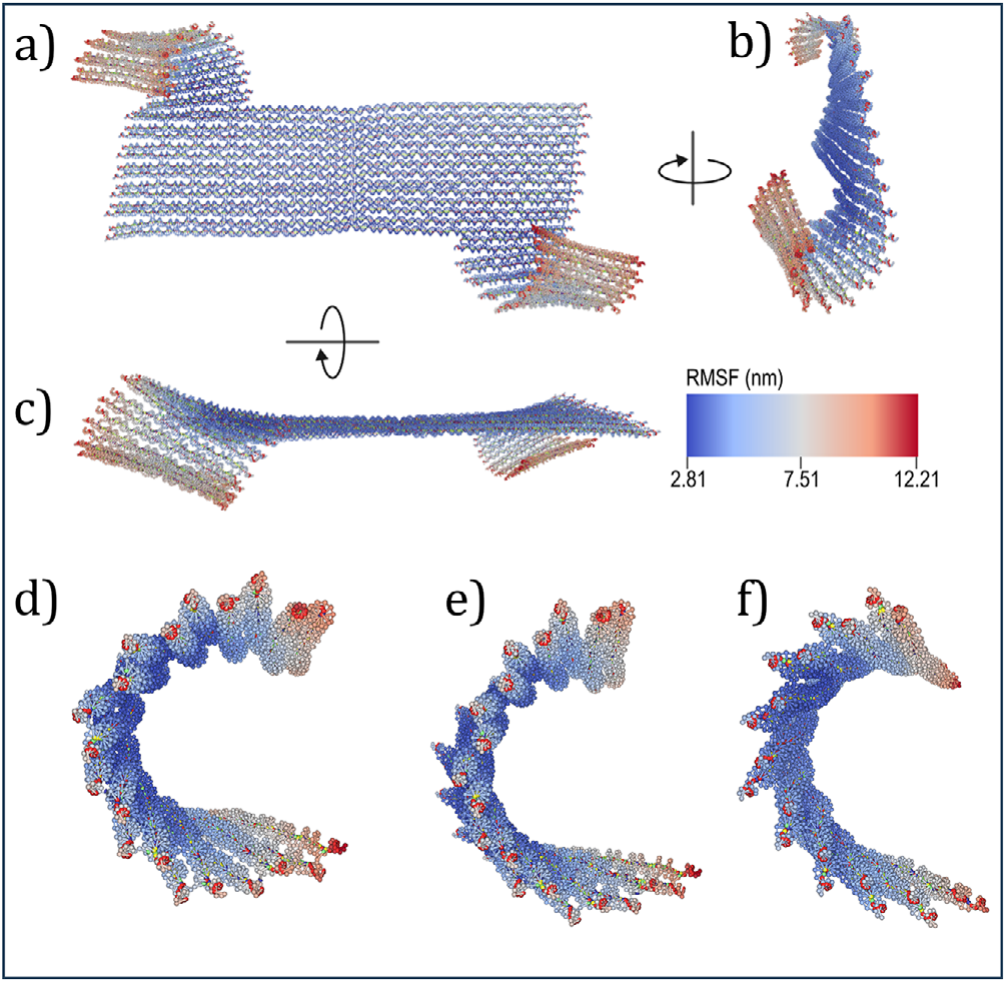
Conformation of the **CDL** DON derived from the mean structure of oxDNA simulations at an ionic strength of 0.5 M Na^+^. Although the DON is designed with a honeycomb lattice, simulations reveal a significant global twist, particularly in the two arms. a) Front view, b) side view, and c) top view. The color of each nucleotide represents its root‐mean‐square fluctuation (RMSF), with the corresponding colormap shown in the bottom‐right corner. (d‐f) Side views of the mean structures calculated from oxDNA simulations of one arm of the **CDL** DON at d) 0.5 M, e) 2.0 M, and f) 5.0 M ionic strength of Na^+^.

In conclusion, this study demonstrates a simple yet effective method for controlling the orientation of **CDL** DON on mica surfaces by tuning the Mg^2^⁺ concentration. Using AFM at both buffer/mica and air/mica interfaces, we observed a distinct shift in **CDL** orientations as a function of Mg^2^⁺ concentration. Most importantly, we demonstrate that a chiral surface featuring only **S** orientations of the adsorbed **CDL** DON can be obtained both in liquid and dry conditions at low Mg^2+^ concentrations below 10 mM. At higher concentrations around 100 mM Mg^2+^, a fully achiral surface (50:50 ratio of **S** and **Z** orientations) is obtained in liquid. However, washing and drying induces **Z**‐shaped DON desorption leading to an increased ratio of about 61:39.

These findings offer valuable insights into the complex interplay of electrostatic forces, surface interactions, and DON conformation. The simplicity of our approach, exploiting Mg^2^⁺ induced shape alterations in 2D DON provides a powerful and accessible method for controlling DON orientation on mica. This strategy bypasses the need for complex DON redesign or surface functionalization, opening new avenues for scalable and cost‐effective nanofabrication.

Beyond fundamental understanding, this controlled orientation strategy has significant implications for various applications. For instance, flat 2D DON such as rectangular and triangular DON have recently been used for data storage^[^ ^29^, ^30^ ^]^ and drug discovery^[^ ^31^, ^32^ ^]^ applications. In both cases, however, it is challenging to control the adsorption orientation of the rectangular and triangular DON. To improve data readout, future studies will utilize chiral DON (**CDL**) to precisely control the adsorption orientation. In general, precise orientation is crucial for the integration of DON with other nano‐materials, such as nanoparticles or quantum dots, for creating nanostructures surfaces with applications in plasmonic, metamaterials, biosensors, and interfaces.

## Supporting Information

The authors have cited additional references within the Supporting Information.^[^ ^1^, ^2^, ^3^, ^4^ ^]^

## Conflict of Interests

The authors declare no conflict of interest.

## Supporting information



Supporting Information

## Data Availability

The data that support the findings of this study are available from the corresponding author upon reasonable request.
