# Supplementary material for: Breaking of the Up‐Down Symmetry of DNA Origami on a Solid Substrate
Source: Angew Chem Int Ed Engl. 2025 Oct 12;64(48):e202507613. doi: 10.1002/anie.202507613 (PMC12643347; doi:10.1002/anie.202507613)
Supplement: Supplementary file 1 — Supporting Information [file ANIE-64-e202507613-s001.pdf]

SUPPORTING INFORMATION

Breaking of the Up-Down Symmetry of DNA Origami on a Solid Substrate

Gangamallaiah Velpula,[a],* Emilia Tomm,[b] Boxuan Shen,[c] Kunal S. Mali,[a] Adrian Keller[b],* and Steven De Feyter[a],*

[a] Dr. Gangamallaiah Velpula, Dr. Kunal S. Mali and Prof. Steven De Feyter
Department of Chemistry, Division of Molecular Imaging and Photonics, KU Leuven, B-3001 Leuven, Belgium

[b] Emilia Tomm, PD Dr. Adrian Keller
Paderborn University, Technical and Macromolecular Chemistry, Warburger Str.100, Paderborn 33098, Germany

[c] Dr. Boxuan Shen
Department of Medical Biochemistry and Biophysics, Karolinska Institutet, 17177 Stockholm, Sweden

E-mail: gm.velpula@kuleuven.be, adrian.keller@uni-paderborn.de, steven.defeyter@kuleuven.be

Table of Contents:

1. **Experimental details**: DNA origami assembly, AFM (AFM) imaging at the air/mica interface and AFM imaging at the buffer/mica interface.
2. OxDNA simulations
3. Large-scale AFM images of CDL DNA origami at the 1x TAE buffer/mica interface containing **3.5 mM Mg^{2+} (Figure S1)**
4. Number of individual, total number and % of S and Z orientations of CDL DNA origami obtained from Figure S1 **(Table S1)**
5. Large-scale AFM images of CDL DNA origami at the 1x TAE buffer/mica interface containing **5.0 mM Mg^{2+} (Figure S2)**
6. Number of individual, total number and % of S and Z orientations of CDL DNA origami obtained from Figure S2 **(Table S2)**
7. Large-scale AFM images of CDL DNA origami at the 1x TAE buffer/mica interface containing **7.5 mM Mg^{2+} (Figure S3)**
8. Number of individual, total number and % of S and Z orientations of CDL DNA origami obtained from Figure S3 **(Table S3)**
9. Large-scale AFM images of CDL DNA origami at the 1x TAE buffer/mica interface containing **10.0 mM Mg^{2+}. (Figure S4)**
10. Number of individual, total number and % of S and Z orientations of CDL DNA origami obtained from Figure S4 **(Table S4)**
11. Large-scale AFM images of CDL DNA origami at the 1x TAE buffer/mica interface containing **25.0 mM Mg^{2+} (Figure S5)**
12. Number of individual, total number and % of S and Z orientations of CDL DNA origami obtained from Figure S5**. (Table S5)**
13. Large-scale AFM images of CDL DNA origami at the 1x TAE buffer/mica interface containing **50.0 mM Mg^{2+} (Figure S6)**
14. Number of individual, total number and % of S and Z orientations of CDL DNA origami obtained from Figure S6 **(Table S6)**
15. Large-scale AFM images of CDL DNA origami at the 1x TAE buffer/mica interface containing **75.0 mM Mg^{2+} (Figure S7)**
16. Number of individual, total number and % of S and Z orientations of CDL DNA origami obtained from Figure S7 **(Table S7)**

17. Large-scale AFM images of CDL DNA origami at the 1x TAE buffer/mica interface containing **100.0 mM Mg^{2+} (Figure S8)**
18. Number of individual, total number and % of S and Z orientations of CDL DNA origami obtained from Figure S8 **(Table S8)**
19. Large-scale AFM images of CDL DNA origami at the air/mica interface containing **3.5 mM Mg^{2+}**. **(Figure S9)**
20. Number of individual, total number and % of S and Z orientations of CDL DNA origami obtained from Figure S9 **(Table S9)**
21. Large-scale AFM images of CDL DNA origami at the air/mica interface containing **5.0 mM Mg^{2+} (Figure S10)**
22. Number of individual, total number and % of S and Z orientations of CDL DNA origami obtained from Figure S10**. (Table S10)**
23. Large-scale AFM images of CDL DNA origami at the air/mica interface containing **7.5 mM Mg^{2+} (Figure S11)**
24. Number of individual, total number and % of S and Z orientations of CDL DNA origami obtained from Figure S11 **(Table S11)**
25. Large-scale AFM images of CDL DNA origami at the air/mica interface containing **10.0 mM Mg^{2+} (Figure S12)**
26. Number of individual, total number and % of S and Z orientations of CDL DNA origami obtained from Figure S12 **(Table S12)**
27. Large-scale AFM images of CDL DNA origami at the air/mica interface containing 2**5.0 mM Mg^{2+}**. **(Figure S13)**
28. Number of individual, total number and % of S and Z orientations of CDL DNA origami obtained from Figure S13 **(Table S13)**
29. Large-scale AFM images of CDL DNA origami at the air/mica interface containing **50.0 mM Mg^{2+} (Figure S14)**
30. Number of individual, total number and % of S and Z orientations of CDL DNA origami obtained from Figure S14 **(Table S14)**
31. Large-scale AFM images of CDL DNA origami at the air/mica interface containing 7**5.0 mM Mg^{2+} (Figure S15)**
32. Number of individual, total number and % of S and Z orientations of CDL DNA origami obtained from Figure S15 **(Table S15)**

33. Large-scale AFM images of CDL DNA origami at the air/mica interface containing **100.0 mM Mg^{2+} (Figure S16)**
34. Number of individual, total number and % of S and Z orientations of CDL DNA origami obtained from Figure S16 **(Table S16)**
35. Control experiment: Large-scale AFM images of CDL DNA origami at the buffer/mica interface containing **100.0 mM Mg^{2+} (Figure S17)**
36. Number of individual, total number and % of S and Z orientations of CDL DNA origami obtained from Figure S17 **(Table S17)**
37. Large-scale AFM images of CDL DNA origami at the air/mica interface containing **100.0 mM Mg^{2+} (Figure S18)**
38. Number of individual, total number and % of S and Z orientations of CDL DNA origami obtained from Figure S18 **(Table S18)**
39. Small-scale AFM images of CDL DNA origami at the 1x TAE buffer/mica interface containing **7.5 mM Mg^{2+} (Figure S19)**
40. Total number of origami's (S+Z) per square micrometre as a function of Mg^{2+} concentration **(Figure S20)**
41. Design aspects of CDL DNA origami **(Figure S21)**
42. Quantifying the curvature of CDL DNA Origami arms (**Figure S22**)

Experimental Details:

DNA Origami Assembly.

The CaDNAno design and sequences of the **CDL** were reported previously.[1] Chiral double-L (CDL) origamis were assembled as described previously[1] by folding the M13mp18 scaffold (Tilibit) via hybridization to 220 staple strands (Eurofin). The staples scaffold mixture was annealed in 1× TAE (Tris base, acetic acid, EDTA) buffer (Thermofisher) containing 10 mM $MgCl_2$ (Sigma-Aldrich) using a thermocycler Primus 25 advanced (VWR). The folded CDL DNA origami were purified by spin-filtering using Amicon Ultra filters with 100 kDa molecular weight cut-off (MWCO) (Millipore). DNA origami concentrations were determined by UV–vis absorption measurements using an BioDrop NanoPhotometer.

AFM imaging at buffer/mica interface

A 40 µL of 0.55 nM (For $[Mg^{2+}]$ ≤5 mM, [CDL] = 1.1 nM) CDL DNA origami solution with 1x TAE (with different Mg^{2+} concentrations) was deposited onto a freshly cleaved mica substrate and subsequently imaged using AFM at the buffer/mica interface. The samples were imaged in tapping mode on a Cypher AFM (Oxford Instruments) using a BLAC40TS probe (spring constant k = 0.1 N/m, resonant frequency f = 110 kHz). Images were acquired with a scan size varying from of 200 nm to 2 µm with a resolution of 512 × 512 pixels and scan rate 3Hz. Images were taken at more than two locations on each sample, with each location separated by at least a few micrometers. The images were analyzed using Scanning Probe Image Processor (SPIP) software.

AFM imaging at the air/mica interface

A 10 µL. of 2.75 nM (For $[Mg^{2+}]$ ≤5 mM, [CDL] = 5.5 nM) CDL DNA origami solution with 1x TAE (with different Mg^{2+} concentrations) was deposited onto a freshly cleaved mica (Agar scientific, G250-1) substrate. After 1 minute, the droplet was removed, and the surface was rinsed with water (high purity ICP-MS grade, pico-pure plus). The mica surface was then dried with compressed air and characterized using AFM at the air/mica interface.

The samples were imaged using tapping mode AFM on a Cypher AFM (Oxford Instruments) with an SCANASYST-AIR probe (spring constant: k = 0.4 N/m, resonant frequency: f = 70 kHz). Images were acquired with a scan size varying from 200 nm to 2 µm with a resolution of 512 × 512 pixels and line rate 1Hz. Images were taken at different locations on each sample, with each location separated by

at least a few micrometers. The images were analyzed using Scanning Probe Image Processor (SPIP) software.

OxDNA simulations:

The caDNAno design of CDL was converted to oxDNA format using tacoxDNA.[2] The oxDNA simulation started with a relaxation of 1×10^5 steps for removing possible overlapping nucleotides, followed by a molecular dynamics simulation run of 1×10^6 steps to reduce overstretched bonds. Afterward, the structure was simulated for 1×10^8 steps, with a time step of 0.005 oxDNA time units. The main simulations were run with the oxDNA[2] model with a salt concentration of 0.5 M, 2.0 M and 5.0 M and a John thermostat at 30 °C. Every 2×10^4 steps were saved as one simulation state. The mean structure shown in Figure 5 was calculated using the oxDNA analysis tool and visualized with oxView.[3, 4]

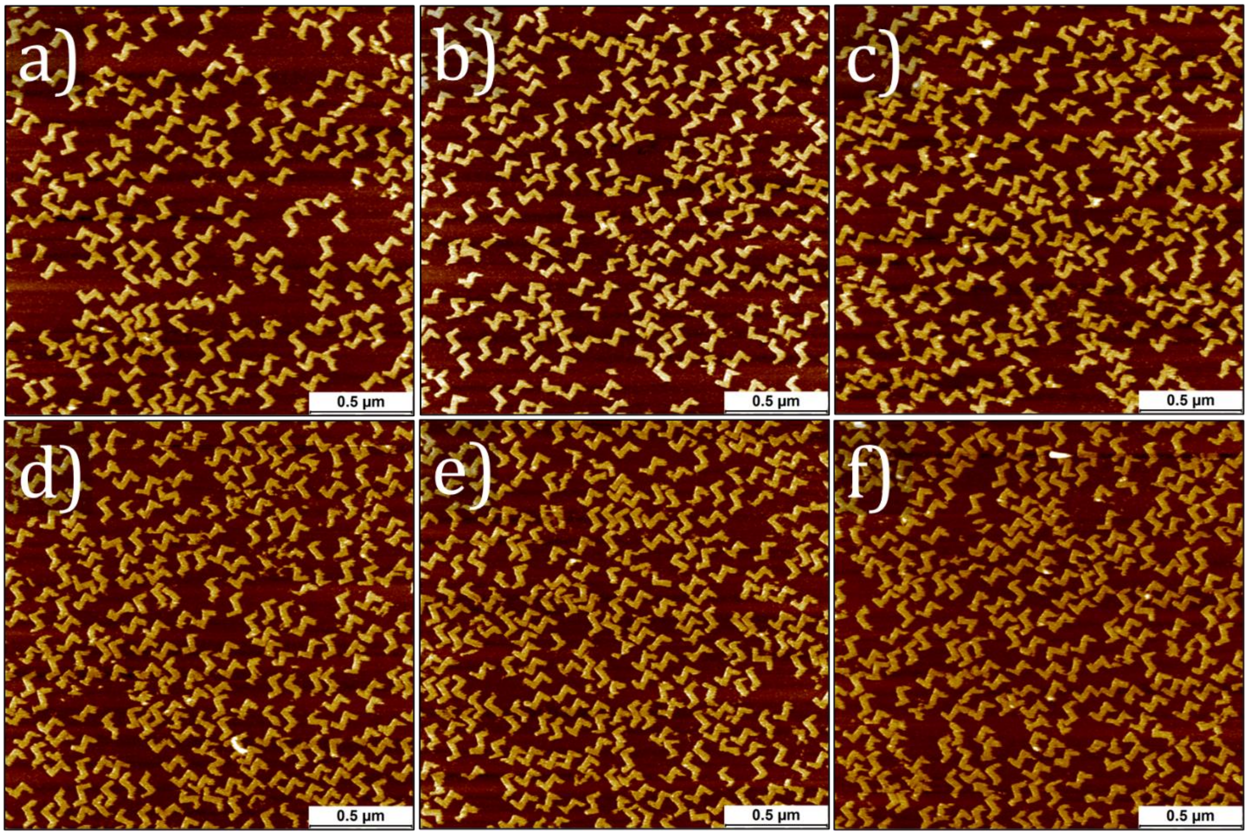

Figure S1. Large-scale AFM images of CDL DNA origami at the 1x TAE buffer/mica interface containing **3.5 mM Mg^{2+}**.

Table S1. Number of individual, total number and % of S and Z orientations of CDL DNA origami obtained from **Figure S1.**

**3.5 mM Mg^{2+}**					
**Image**	**S**	**Z**	**(S+Z)**	**% of S**	**% of Z**
a	134	1	135	99.3	0.7
b	164	5	169	97.0	3.0
c	159	2	161	98.9	1.2
d	176	4	180	97.8	2.2
e	185	5	190	97.4	2.6
f	187	3	190	98.4	1.6
**Total**	**1005**	**20**	**1025**	**98.1**	**1.9**

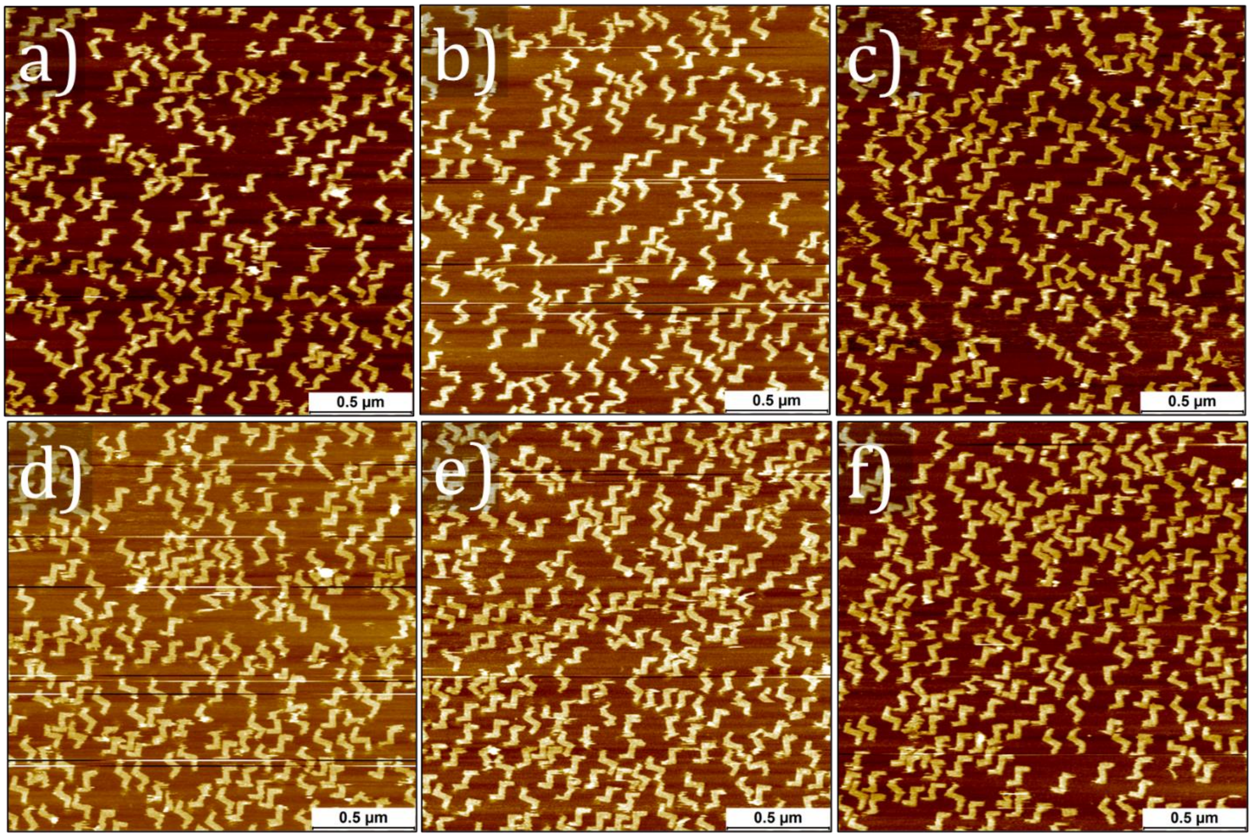

Figure S2. Large-scale AFM images of CDL DNA origami at the 1x TAE buffer/mica interface containing **5.0 mM Mg^{2+}**.

Table S2. Number of individual, total number and % of S and Z orientations of CDL DNA origami obtained from **Figure S2.**

**5.0 mM Mg^{2+}**					
**Sl. No.**	**S**	**Z**	**(S+Z)**	**% of S**	**% of Z**
1	180	1	181	99.4	0.6
2	140	0	140	100	0
3	169	0	169	100	0
4	127	0	127	100	0
5	158	2	160	98.8	1.2
6	180	2	182	98.9	1.1
**Total**	**954**	**5**	**959**	**99.5**	**0.5**

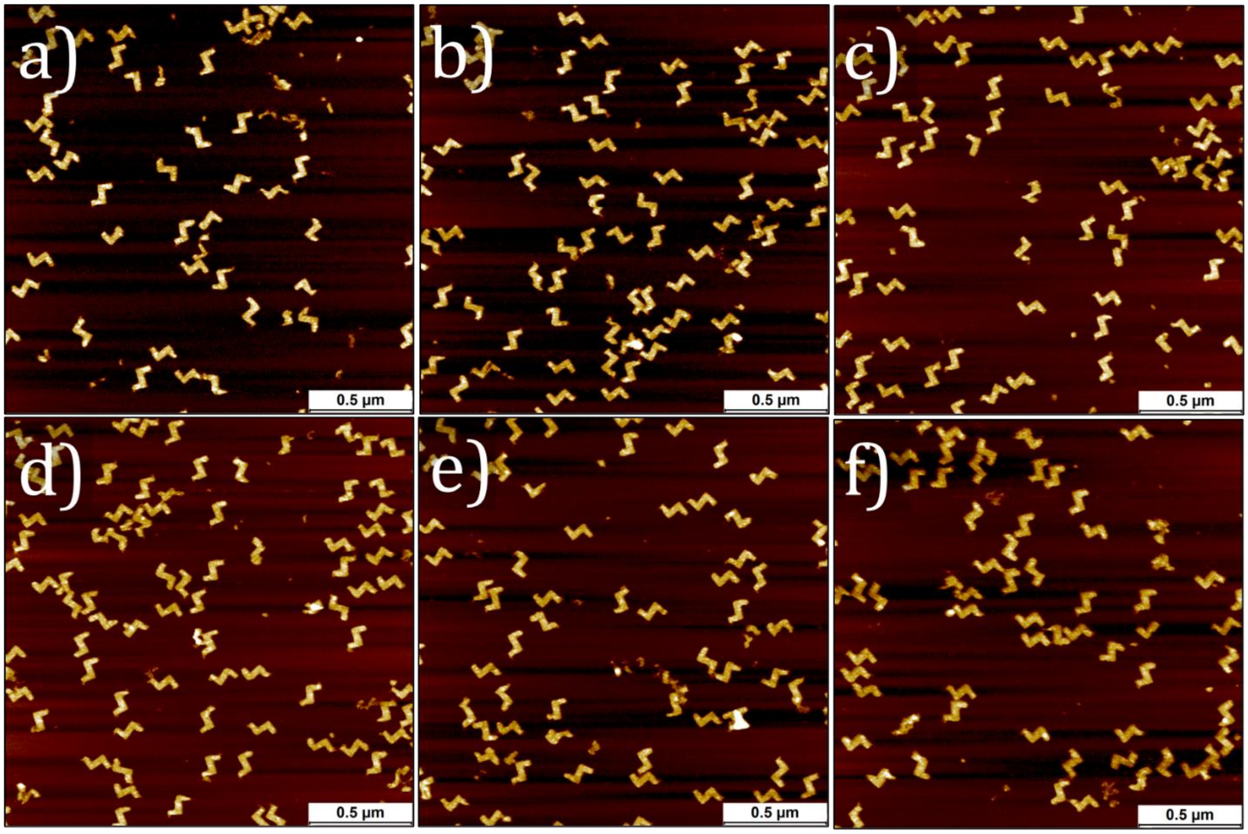

Figure S3. Large-scale AFM images of CDL DNA origami at the 1x TAE buffer/mica interface containing **7.5 mM Mg^{2+}**.

Table S3. Number of individual, total number and % of S and Z orientations of CDL DNA origami obtained from **Figure S3.**

**7.5 mM Mg^{2+}**					
**Sl. No.**	**S**	**Z**	**(S+Z)**	**% of S**	**% of Z**
1	37	6	43	86.0	14.0
2	60	9	69	87.0	13.0
3	58	7	65	89.2	10.8
4	69	8	77	89.6	10.4
5	48	6	54	88.9	11.1
6	45	9	54	83.7	16.7
**Total**	**317**	**45**	**362**	**87.3**	**12.7**

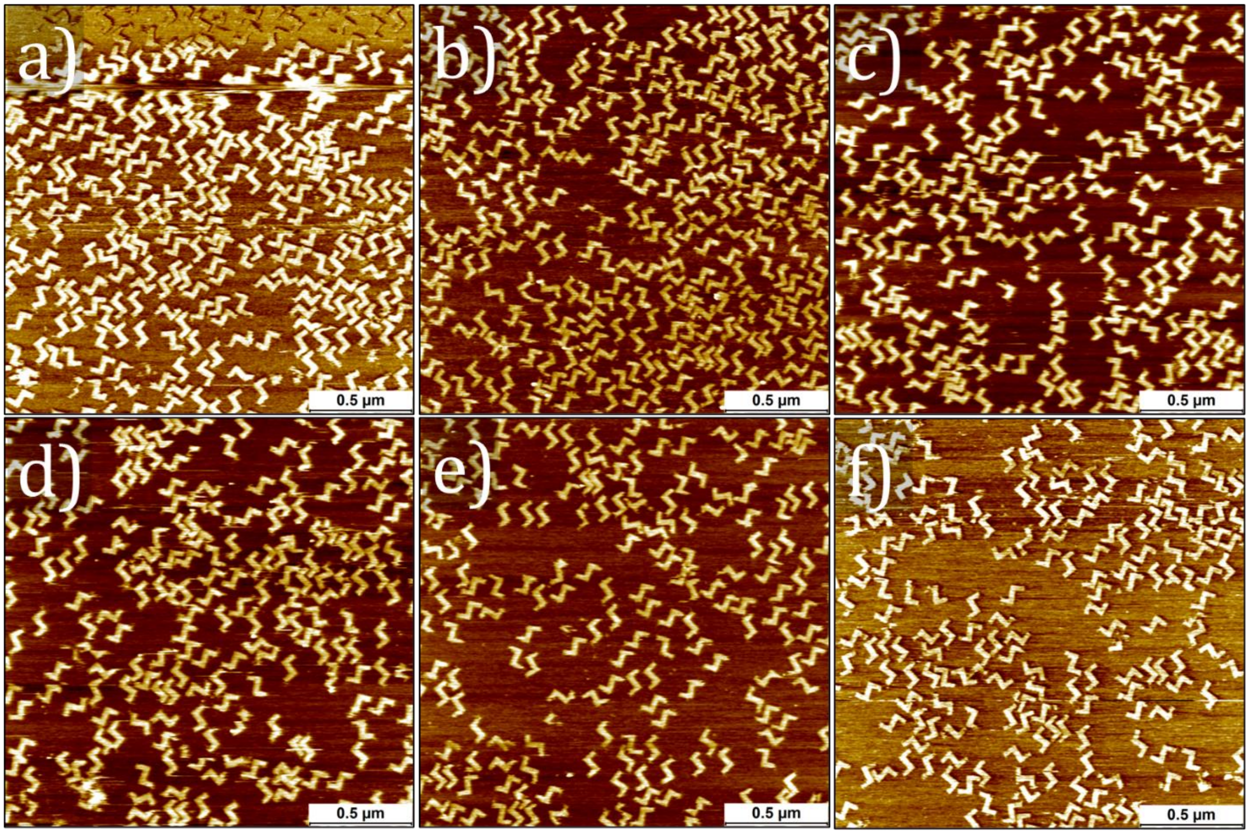

Figure S4. Large-scale AFM images of CDL DNA origami at the 1x TAE buffer/mica interface containing **10.0 mM Mg^{2+}**.

Table 4. Number of individual, total number and % of S and Z orientations of CDL DNA origami obtained from **Figure S4.**

**10.0 mM Mg^{2+}**					
**Sl. No.**	**S**	**Z**	**(S+Z)**	**% of S**	**% of Z**
1	185	42	227	81.5	18.5
2	208	45	253	82.2	17.8
3	144	42	186	77.4	22.6
4	140	27	167	83.8	16.2
5	139	29	168	82.7	17.3
6	134	39	173	77.5	22.5
**Total**	**950**	**224**	**1174**	**80.9**	**19.1**

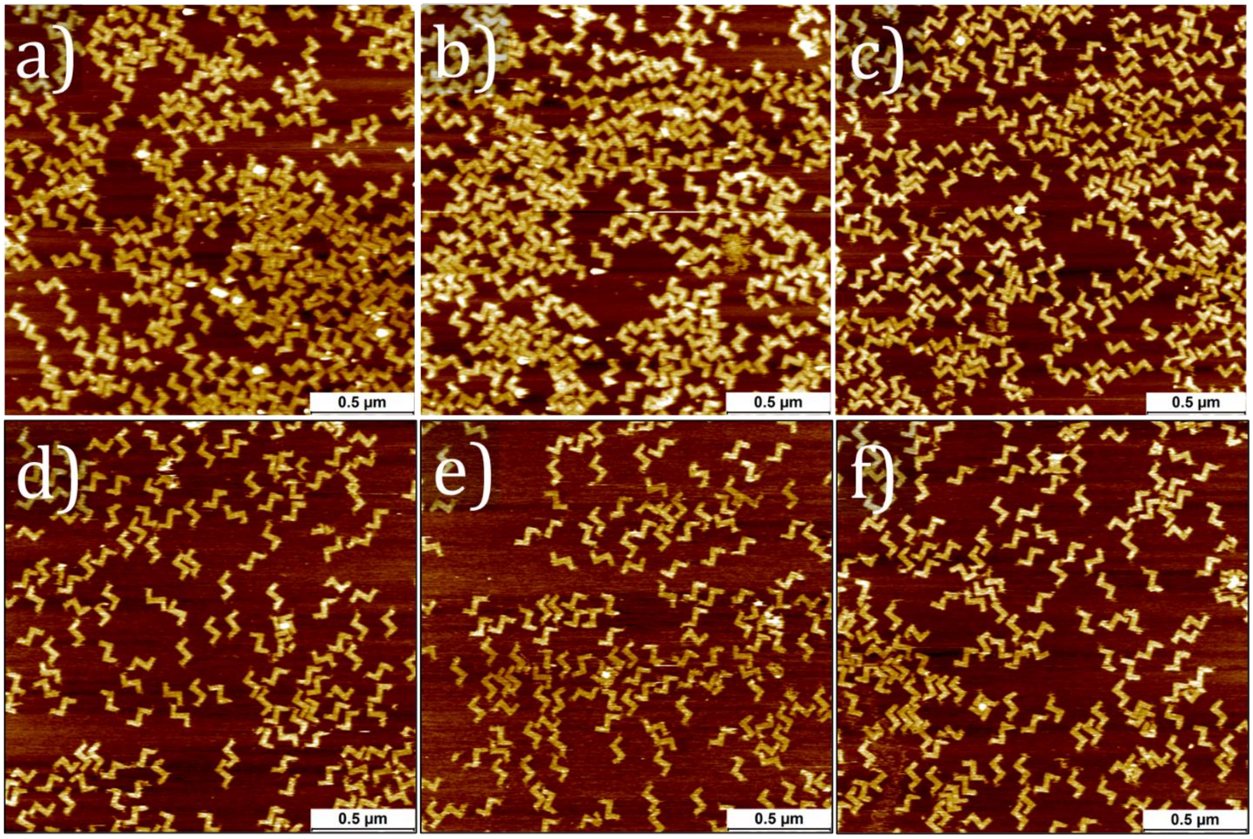

Figure S5. Large-scale AFM images of CDL DNA origami at the 1x TAE buffer/mica interface containing 2**5.0 mM Mg^{2+}**.

Table S5. Number of individual, total number and % of S and Z orientations of CDL DNA origami obtained from **Figure S1.**

**25.0 mM Mg^{2+}**					
**Sl. No.**	**S**	**Z**	**(S+Z)**	**% of S**	**% of Z**
a	157	50	207	75.9	24.1
b	164	54	218	75.2	24.8
c	168	64	232	72.4	27.6
d	134	39	173	77.5	22.5
e	134	39	173	77.5	22.5
f	114	40	154	74.0	26.0
**Total**	**871**	**286**	**1157**	**75.4**	**24.6**

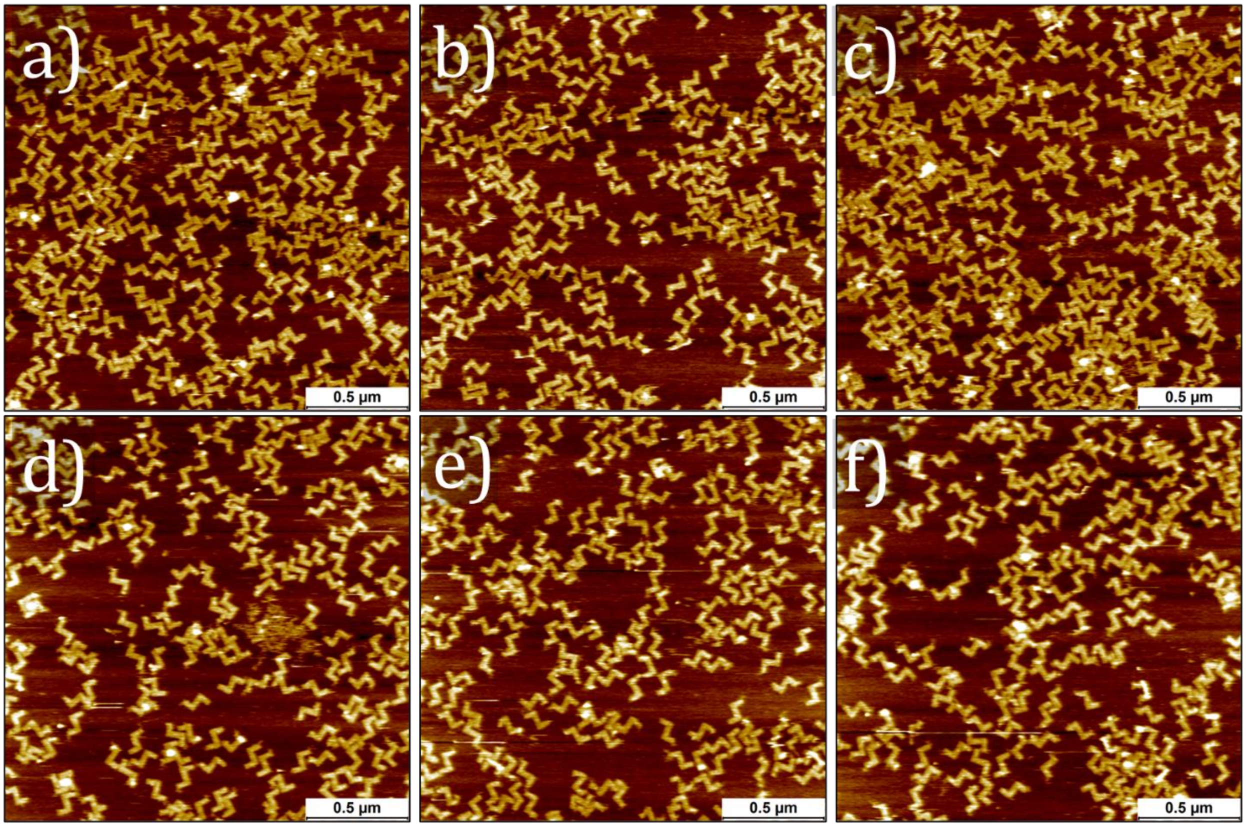

Figure S6. Large-scale AFM images of CDL DNA origami at the 1x TAE buffer/mica interface containing **50.0 mM Mg^{2+}**.

Table S6. Number of individual, total number and % of S and Z orientations of CDL DNA origami obtained from **Figure S6.**

**50.0 mM Mg^{2+}**					
**Sl. No.**	**S**	**Z**	**(S+Z)**	**% of S**	**% of Z**
a	135	78	213	63.4	36.6
b	101	87	188	53.7	46.3
c	148	74	222	66.7	33.3
d	111	49	160	69.4	30.4
e	104	60	164	63.4	36.6
f	103	57	160	64.4	35.6
**Total**	**702**	**405**	**1107**	**63.5**	**36.5**

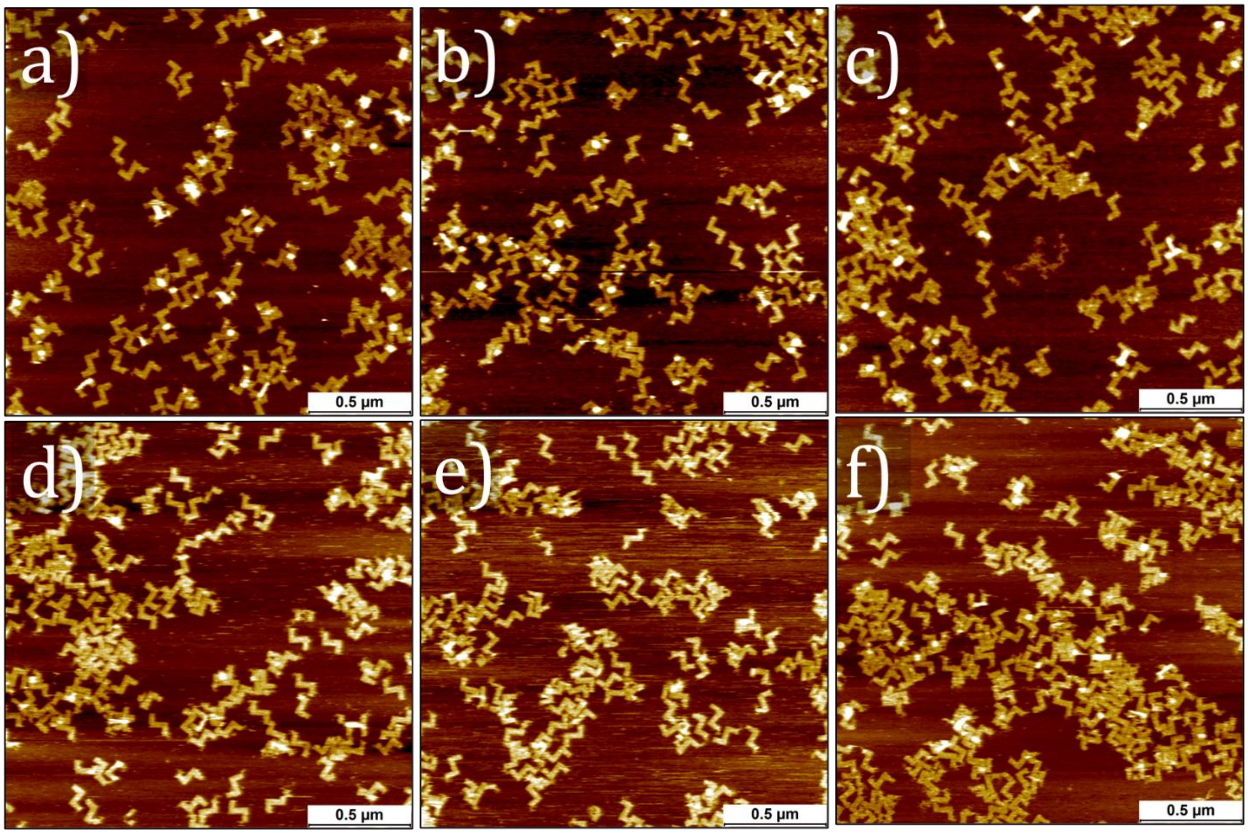

Figure S7. Large-scale AFM images of CDL DNA origami at the 1x TAE buffer/mica interface containing 7**5.0 mM Mg^{2+}**.

Table S7. Number of individual, total number and % of S and Z orientations of CDL DNA origami obtained from **Figure S7.**

**75.0 mM Mg^{2+}**					
**Sl. No.**	**S**	**Z**	**(S+Z)**	**% of S**	**% of Z**
a	53	38	91	58.2	41.8
b	65	40	105	61.9	38.1
c	69	48	117	59.0	41.0
d	42	41	83	50.6	49.4
e	48	37	85	56.5	43.5
f	61	41	102	59.8	40.2
**Total**	**338**	**245**	**583**	**57.7**	**42.3**

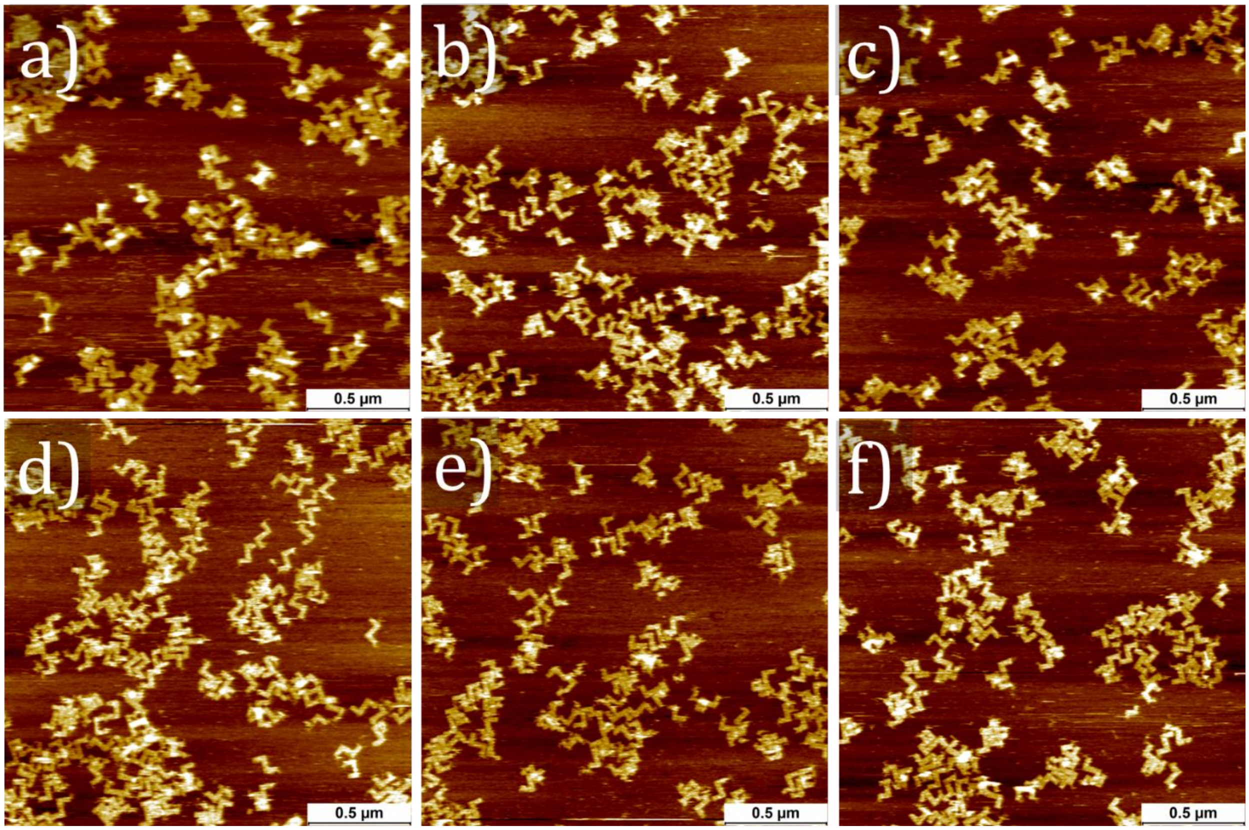

Figure S8. Large-scale AFM images of CDL DNA origami at the 1x TAE buffer/mica interface containing **100.0 mM Mg^{2+}**.

Table S8. Number of individual, total number and % of S and Z orientations of CDL DNA origami obtained from **Figure S8.**

**100.0 mM Mg^{2+}**					
**Sl. No.**	**S**	**Z**	**(S+Z)**	**% of S**	**% of Z**
a	33	29	61	54.1	45.9
b	27	30	57	47.4	52.6
c	26	25	51	51.0	49.0
d	43	42	85	50.6	49.4
e	36	33	69	52.2	47.8
f	41	32	73	56.2	43.8
**Total**	**206**	**191**	**397**	**51.7**	**48.3**

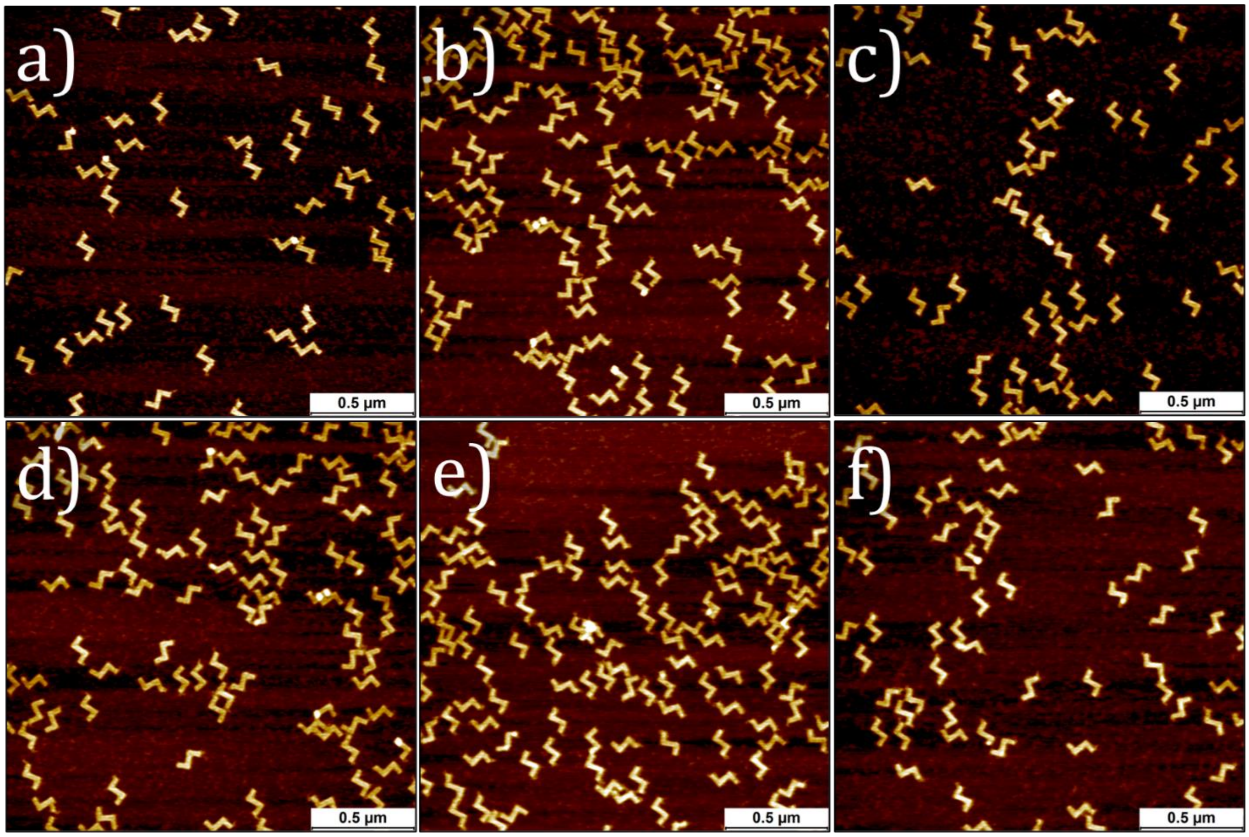

Figure S9. Large-scale AFM images of CDL DNA origami at the air/mica interface containing **3.5 mM Mg^{2+}**.

Table S9. Number of individual, total number and % of S and Z orientations of CDL DNA origami obtained from **Figure S9.**

**3.5 mM Mg^{2+}**					
**Sl. No.**	**S**	**Z**	**(S+Z)**	**% of S**	**% of Z**
1	47	0	47	100.0	0
2	121	1	122	99.2	0.82
3	59	1	60	98.3	1.667
4	100	1	101	99.0	0.99
5	122	1	123	99.2	0.813
6	68	0	68	100.0	0
**Total**	**517**	**4**	**521**	**99.3**	**0.7**

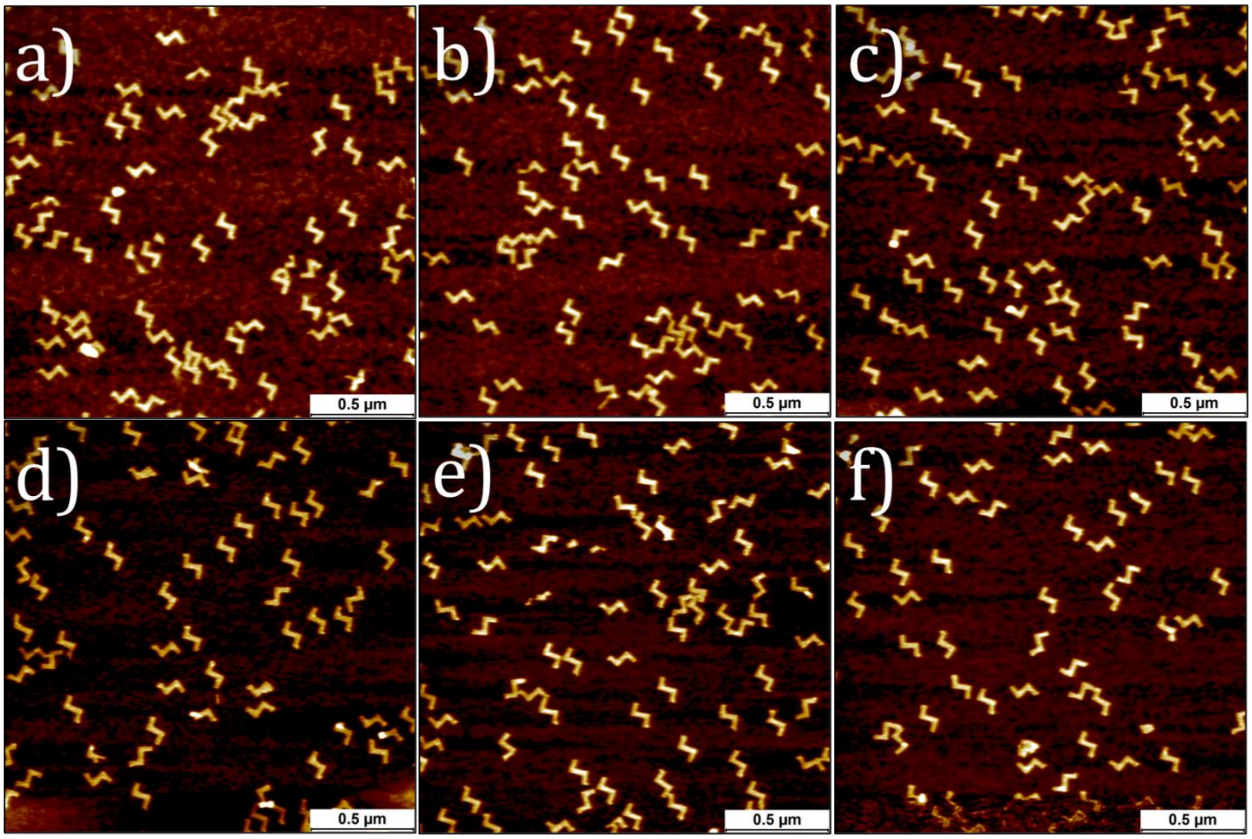

Figure S10. Large-scale AFM images of CDL DNA origami at the air/mica interface containing **5.0 mM Mg^{2+}**.

Table S10. Number of individual, total number and % of S and Z orientations of CDL DNA origami obtained from **Figure S10.**

**5.0 mM Mg^{2+}**					
**Sl. No.**	**S**	**Z**	**(S+Z)**	**% of S**	**% of Z**
1	60	0	60	100	0
2	70	2	72	97.2	2.8
3	64	1	65	98.5	1.5
4	64	1	65	98.5	1.5
5	68	2	70	97.1	2.9
6	57	1	58	98.2	1.7
**Total**	**387**	**8**	**395**	**98.0**	**2.0**

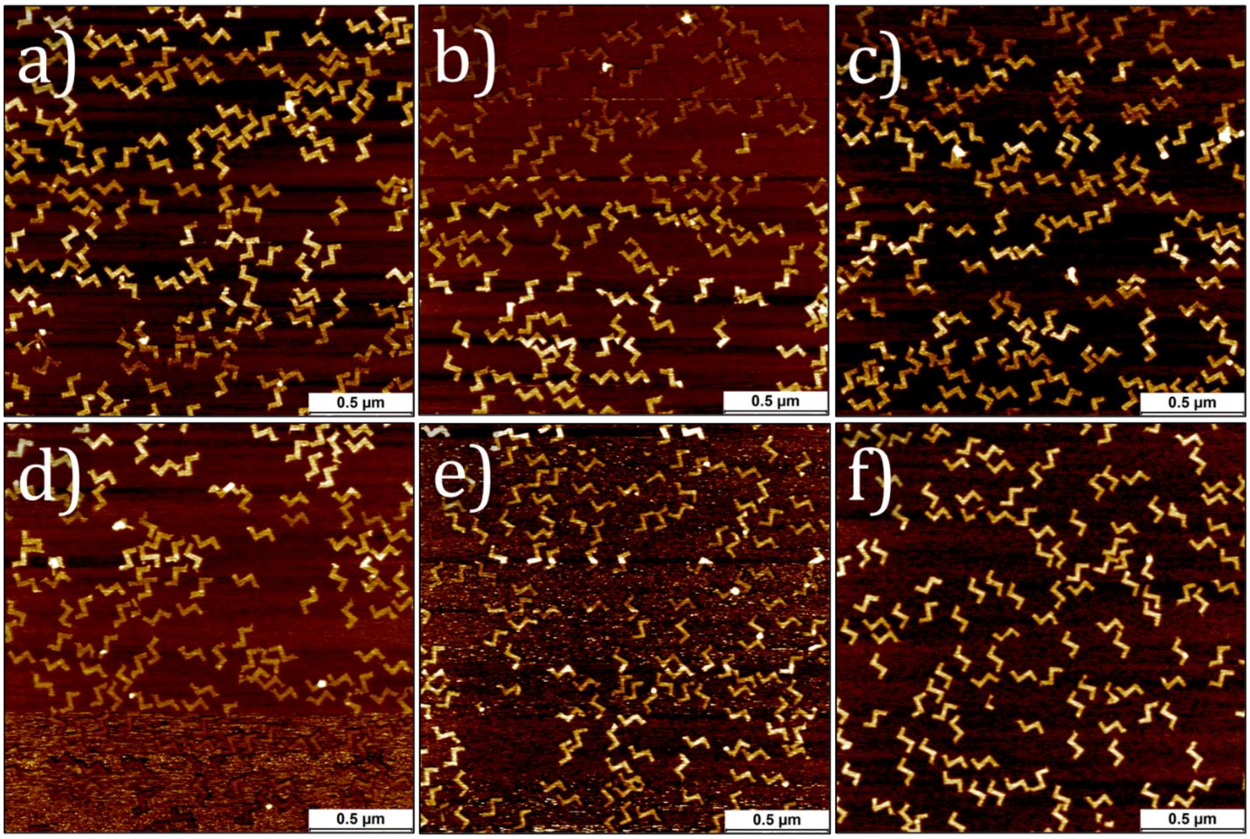

Figure S11. Large-scale AFM images of CDL DNA origami at the air/mica interface containing **7.5.0 mM Mg^{2+}**.

Table S11. Number of individual, total number and % of S and Z orientations of CDL DNA origami obtained from **Figure S11.**

**7.5 mM Mg^{2+}**					
**Sl. No.**	**S**	**Z**	**(S+Z)**	**% of S**	**% of Z**
1	144	7	151	95.4	4.6
2	132	4	136	97.1	2.9
3	146	7	153	95.4	4.6
4	138	6	144	95.8	4.2
5	127	9	136	93.4	6.6
6	116	4	120	96. 7	3.3
**Total**	**803**	**37**	**840**	**95.6**	**4.4**

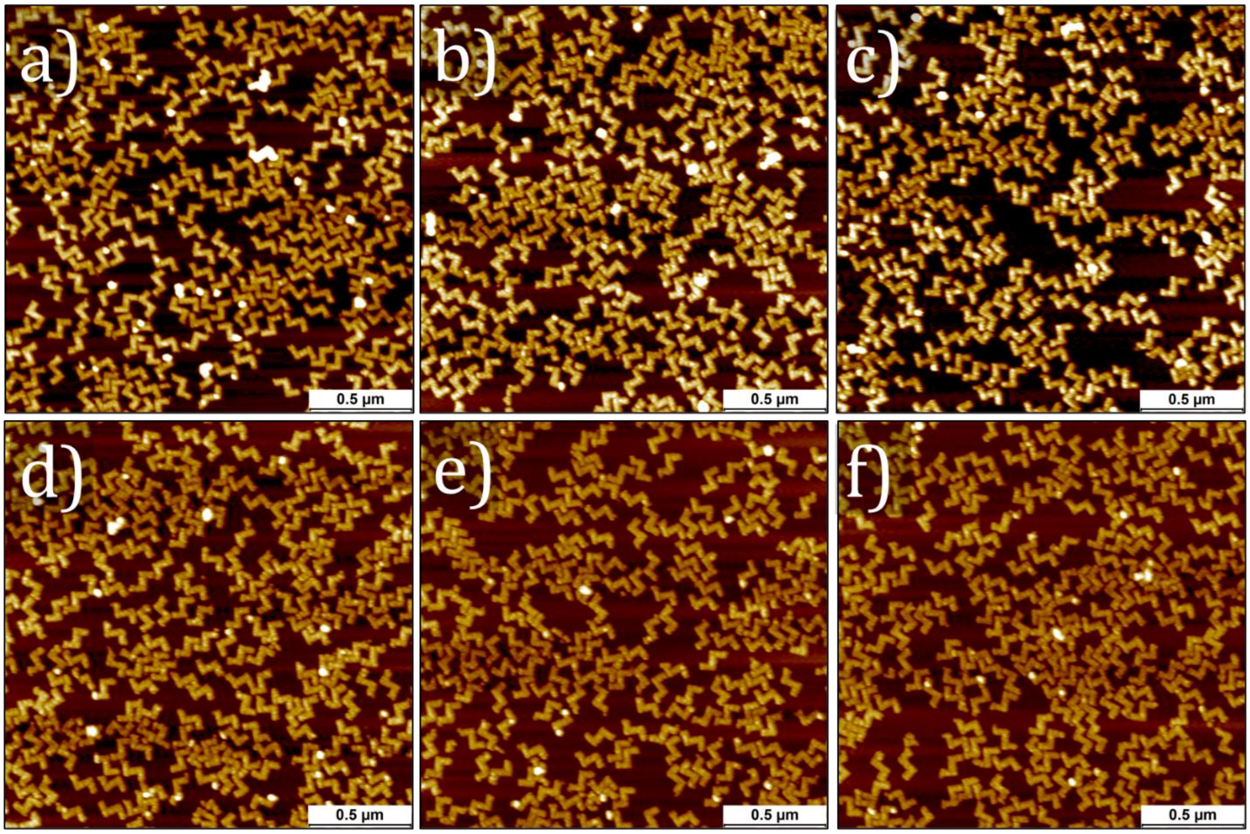

Figure S12. Large-scale AFM images of CDL DNA origami at the air/mica interface containing **10.0 mM Mg^{2+}**.

Table S12. Number of individual, total number and % of S and Z orientations of CDL DNA origami obtained from **Figure S12.**

**10.0 mM Mg^{2+}**					
**Sl. No.**	**S**	**Z**	**(S+Z)**	**% of S**	**% of Z**
1	208	35	243	85.6	14.4
2	210	29	239	87.9	12.1
3	181	28	209	86.6	13.4
4	202	32	234	86.3	13.7
5	192	29	221	86.9	13.1
6	210	31	241	87.1	12.9
**Total**	**1203**	**184**	**1387**	**86.7**	**13.3**

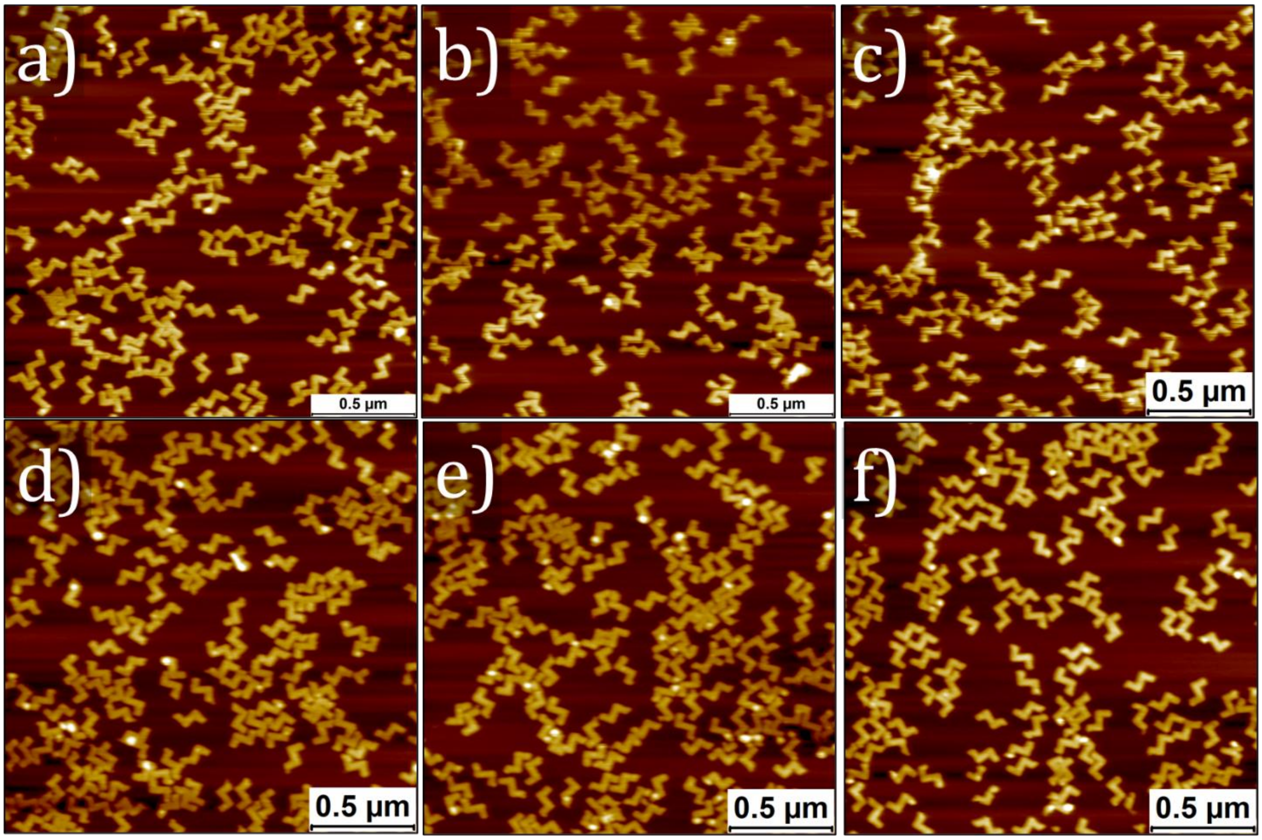

Figure S13. Large-scale AFM images of CDL DNA origami at the air/mica interface containing 2**5.0 mM Mg**$^{2+}$.

Table S13. Number of individual, total number and % of S and Z orientations of CDL DNA origami obtained from **Figure S13.**

**25.0 mM Mg^{2+}**					
**Sl. No.**	**S**	**Z**	**(S+Z)**	**% of S**	**% of Z**
1	102	36	138	73.9	26.1
2	70	37	107	65.4	34.6
3	89	26	115	77.4	22.6
4	111	45	156	71.2	28.8
5	105	41	146	71.9	28.1
6	104	40	144	72.2	27.8
**Total**	**581**	**225**	**806**	**72.0**	**28.0**

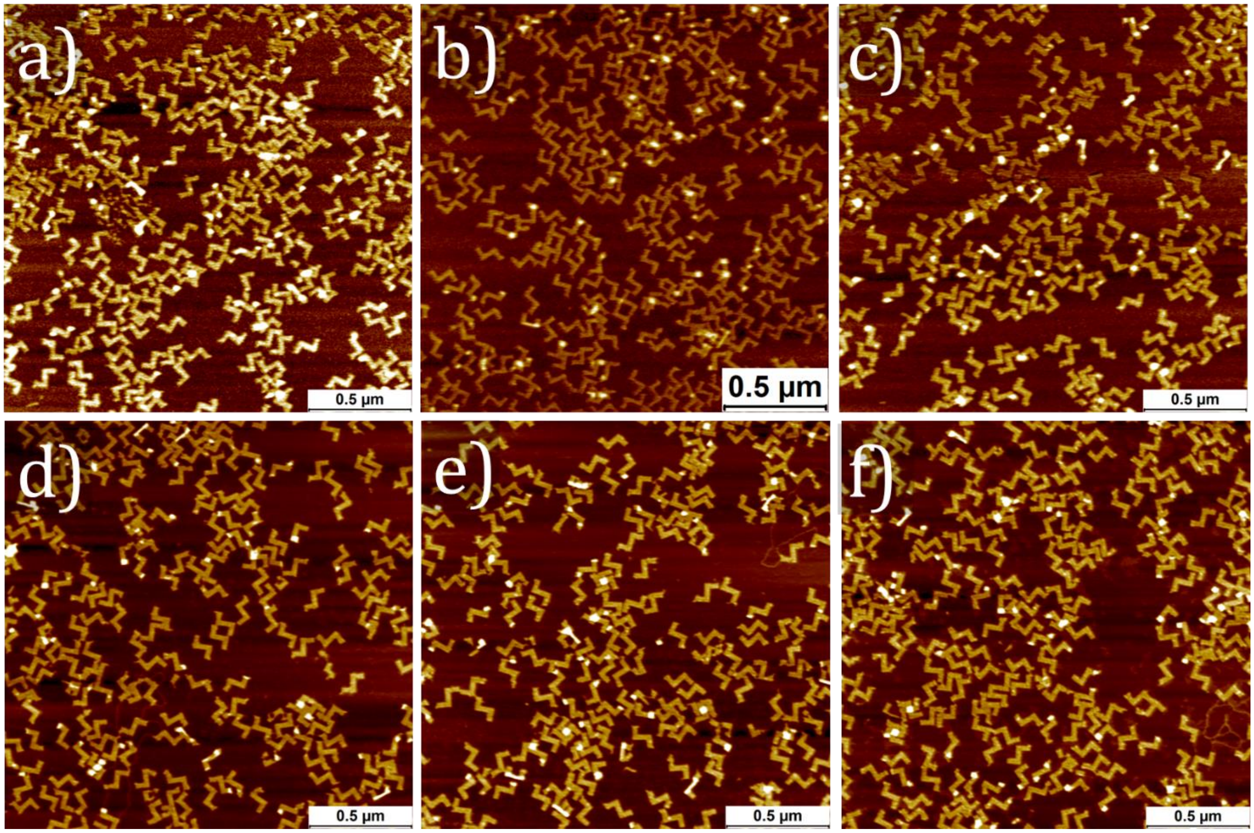

Figure S14. Large-scale AFM images of CDL DNA origami at the air/mica interface containing **50.0 mM Mg^{2+}**.

Table S14. Number of individual, total number and % of S and Z orientations of CDL DNA origami obtained from **Figure S14.**

**50.0 mM Mg^{2+}**					
**Sl. No.**	**S**	**Z**	**(S+Z)**	**% of S**	**% of Z**
1	123	58	181	68.0	32.0
2	138	58	196	70.4	29.6
3	113	61	174	64.9	35.1
4	115	60	175	65.7	34.3
5	110	53	163	67.5	32.5
6	130	54	184	70.7	29.3
**Total**	**729**	**344**	**1073**	**67.9**	**32.1**

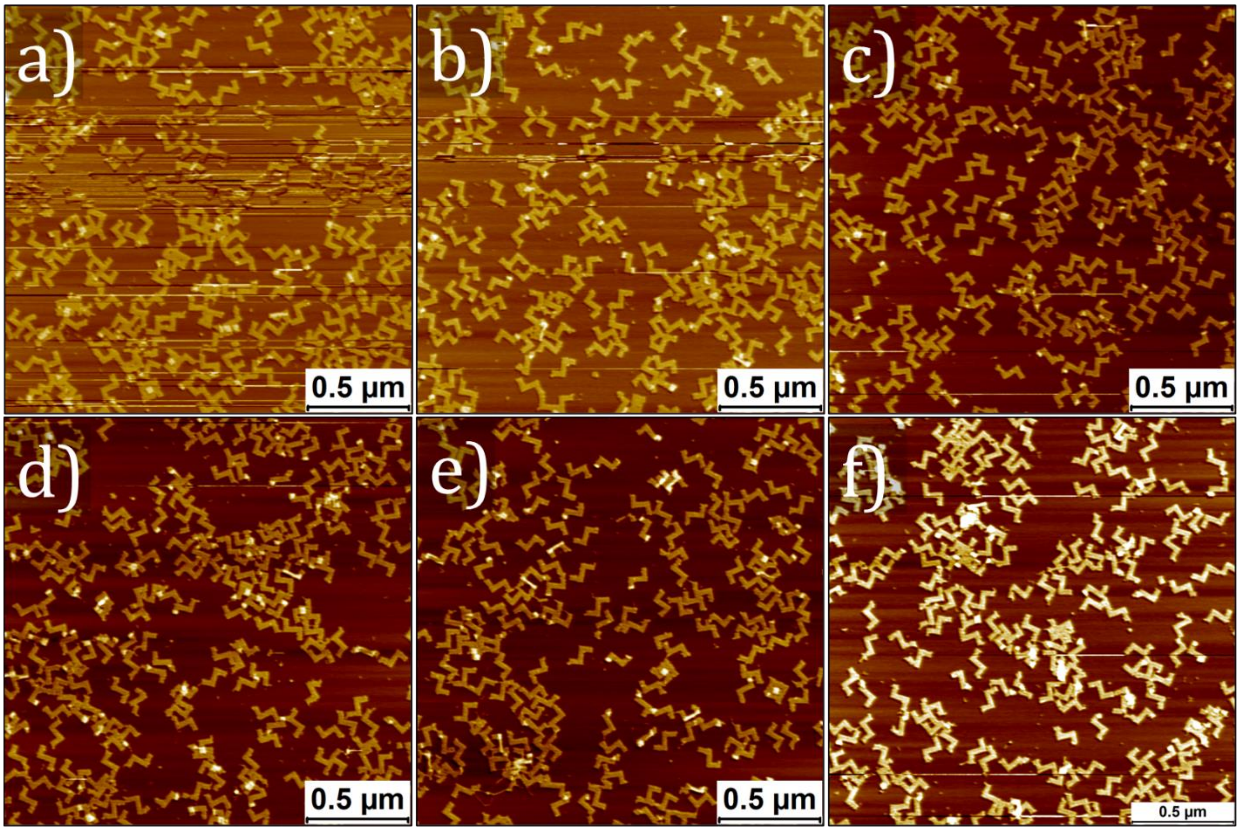

Figure S15. Large-scale AFM images of CDL DNA origami at the air/mica interface containing 7**5.0 mM Mg^{2+}**.

Table S15. Number of individual, total number and % of S and Z orientations of CDL DNA origami obtained from **Figure S15.**

**75.0 mM Mg^{2+}**					
**Sl. No.**	**S**	**Z**	**(S+Z)**	**% of S**	**% of Z**
1	116	51	167	69.5	30.5
2	90	45	135	66.7	33.3
3	71	51	122	58.2	41.8
4	91	44	135	67.4	32.6
5	108	40	148	73.0	27.0
6	94	46	140	67.1	32.9
**Total**	**570**	**277**	**847**	**67.0**	**33.0**

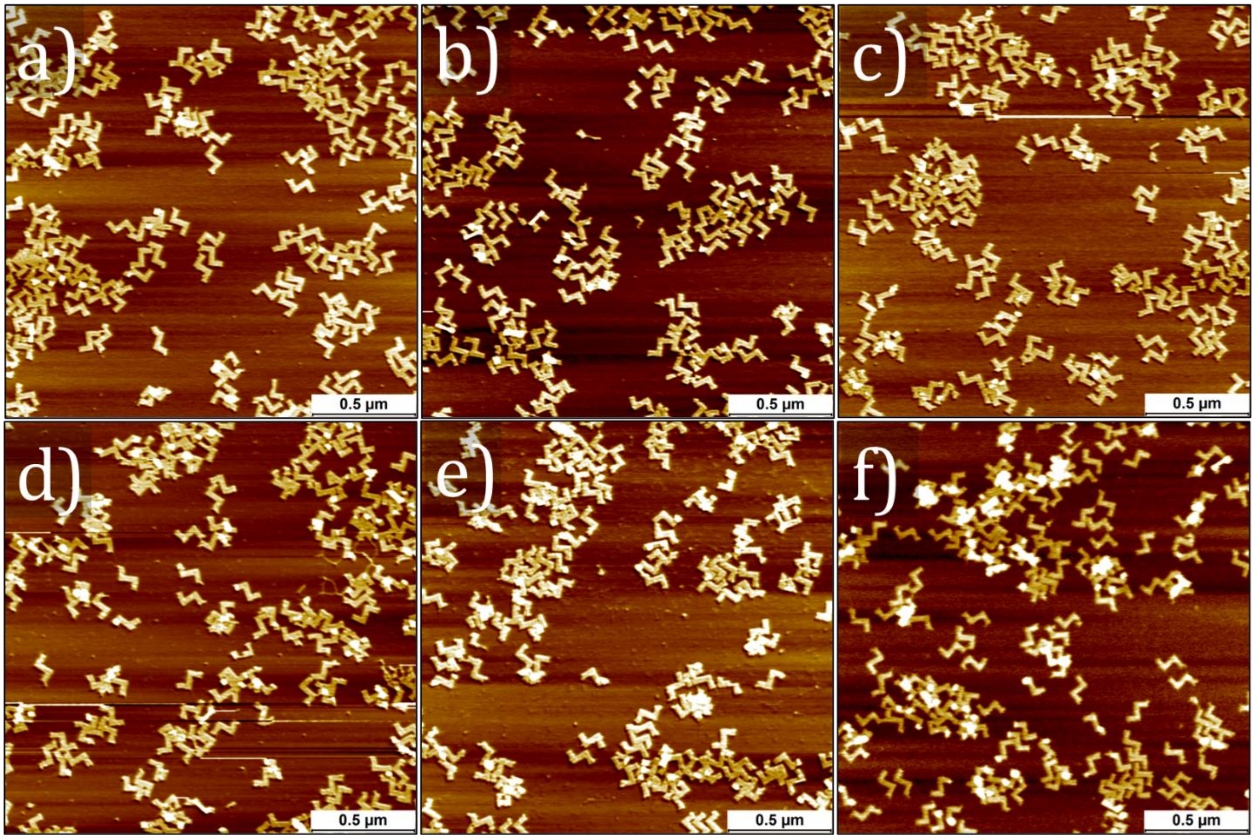

Figure S16. Large-scale AFM images of CDL DNA origami at the air/mica interface containing **100.0 mM Mg^{2+}**.

Table S16. Number of individual, total number and % of S and Z orientations of CDL DNA origami obtained from **Figure S16.**

**100.0 mM Mg^{2+}**					
**Sl. No.**	**S**	**Z**	**(S+Z)**	**% of S**	**% of Z**
1	82	53	135	60.7	39.3
2	63	43	106	59.4	40.6
3	49	22	71	69.0	31.0
4	78	14	92	84.8	15.2
5	75	38	113	66.4	33.6
6	68	22	90	75.6	24.4
**Total**	**415**	**192**	**607**	**69.3**	**30.7**

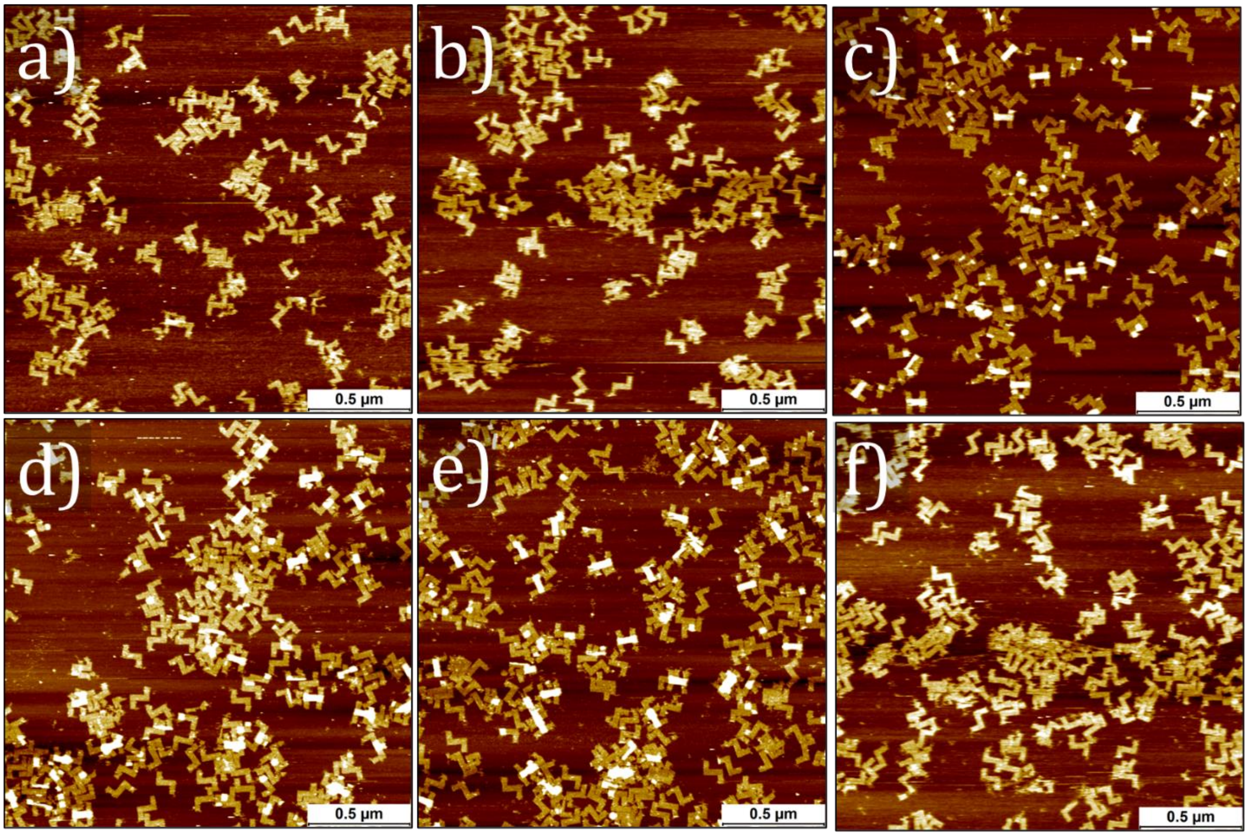

Figure S17. Control Experiment: Large-scale AFM images of CDL DNA origami at the buffer/mica interface containing **100.0 mM Mg^{2+}**.

Table S17. Number of individual, total number and % of S and Z orientations of CDL DNA origami obtained from **Figure S17.**

**100.0 mM Mg^{2+}**					
**Sl. No.**	**S**	**Z**	**(S+Z)**	**% of S**	**% of Z**
1	45	45	90	50	50
2	46	53	99	46.5	53.5
3	53	46	99	53.5	46.5
4	57	46	103	55.3	44.7
5	67	64	131	51.1	48.9
6	64	65	129	49.6	50.4
**Total**	**332**	**319**	**651**	**51.0**	**49.0**

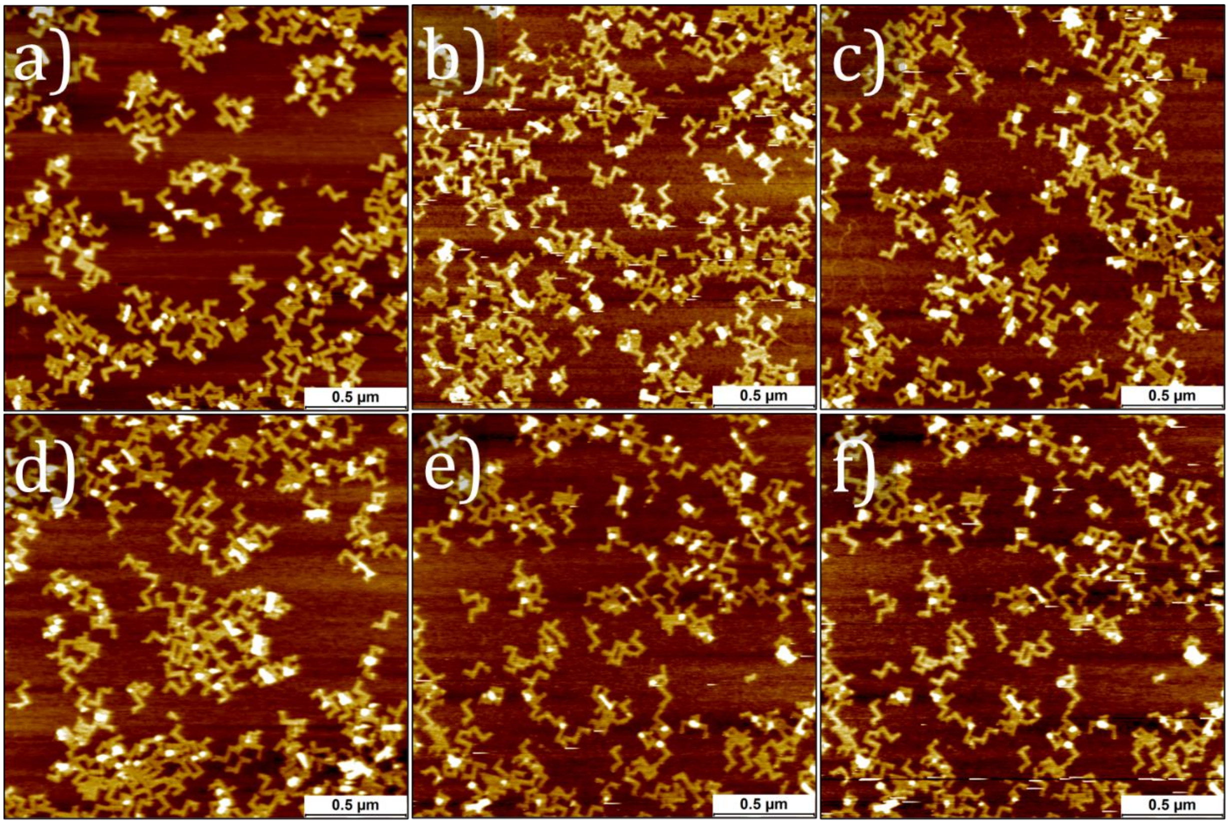

Figure S18. Control Experiment: Large-scale AFM images of CDL DNA origami at the air/mica interface containing **100.0 mM Mg^{2+}**.

Table S18. Number of individual, total number and % of S and Z orientations of CDL DNA origami obtained from **Figure S18.**

**100.0 mM Mg^{2+}**					
**Sl. No.**	**S**	**Z**	**(S+Z)**	**% of S**	**% of Z**
1	54	33	87	62.1	37.9
2	71	45	116	61.2	38.8
3	59	36	95	62.1	37.9
4	48	40	88	54.5	45.5
5	62	36	98	63.3	36.7
6	61	39	100	61.0	39.0
**Total**	**355**	**229**	**584**	**60.7**	**39.3**

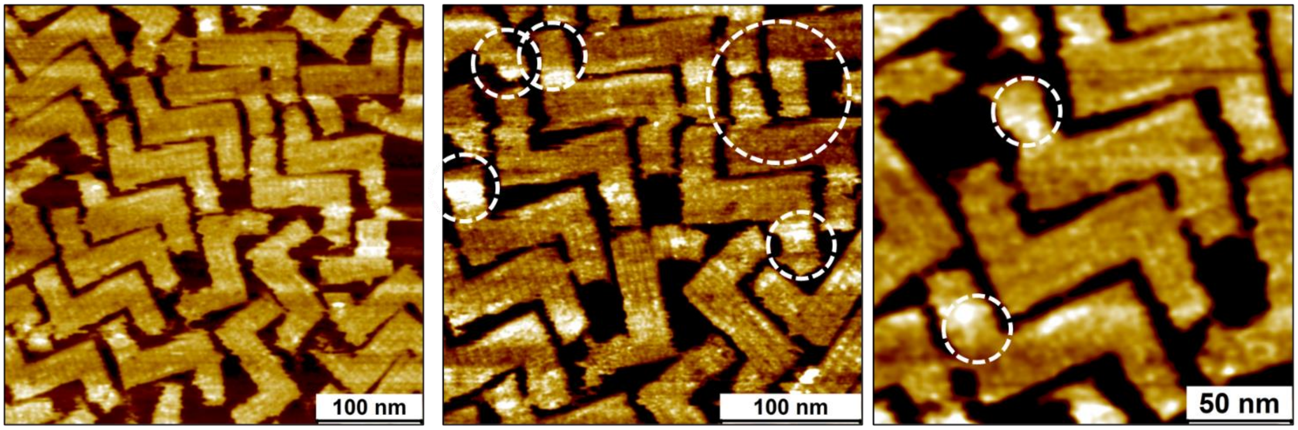

Figure S19. Small-scale AFM images of CDL DNA origami at the 1x TAE buffer/mica interface containing **7.5 mM Mg^{2+}**. White circles show relatively brighter arms on **S** orientation.

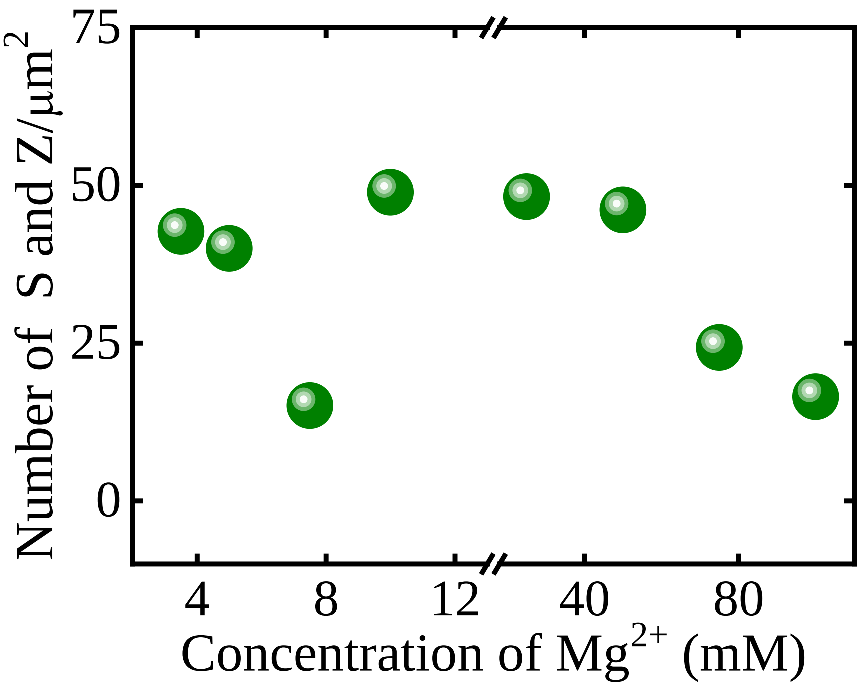

Figure S20. Total number of DNA origami (S+Z) per square micrometre as a function of Mg^{2+} concentration.

Design aspects of CDL DNA origami:

The CDL was originally designed using a native honeycomb lattice with the intention of being "twist-free," as 2D DNA origami structures based on the square lattice exhibit strong intrinsic twist. At the time of design, oxDNA was not yet widely adopted; instead, the standard CanDo simulation tool predicted a perfectly flat geometry. However, recent oxDNA simulations revealed a pronounced curvature in the mean structure. Interestingly, the longer central bar exhibited less curvature compared to the arm regions, which was unexpected. We suspect that the curvature arises from an imbalance in the number of staple crossovers between adjacent helices in the arm regions. Each double crossover (Holliday junction) introduces a local twist, and if the distribution of crossovers is not well balanced, this can result in global bending. To test this, we simulated a single arm of the CDL structure with three different crossover configurations. When all middle crossovers between helices (e.g., between helices 0–1, 2–3, etc.) were removed, the mean structure became nearly straight. Interestingly, adding additional crossovers between adjacent helices (e.g., 1–2, 3–4, etc.) did not produce curvature in the opposite direction.

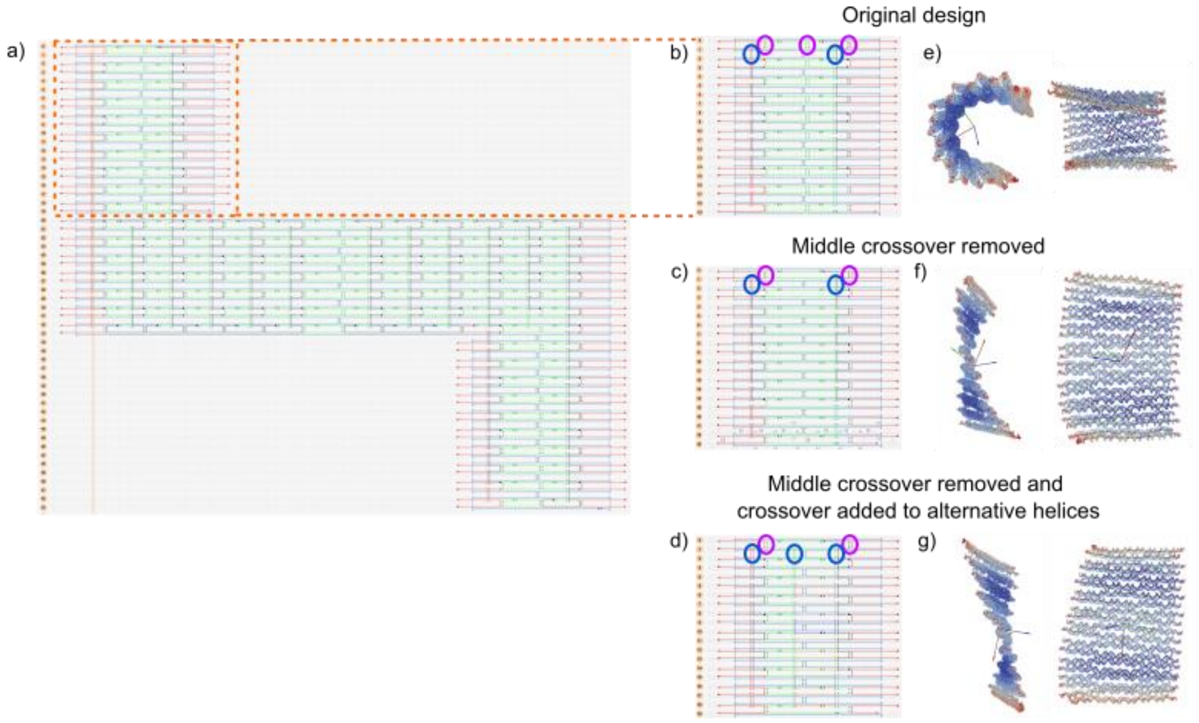

Figure S21. caDNAno designs and oxDNA simulations of one arm of the CDL DNA origami. (a) Full caDNAno schematic of the original CDL design. (b–d) Detailed caDNAno layouts of one arm with alternative crossover patterns. Purple circles mark crossovers connecting even-to-odd helices,

and blue circles mark crossovers connecting odd-to-even helices. (b) Original design. (c) Modified design with the middle even-to-odd crossovers removed. (d) Further modified design based on (c), with additional odd-to-even crossovers introduced. (e–j) Mean structures from oxDNA simulations of the designs in (b–d), shown in side and front views. The different crossover patterns result in distinct arm curvatures, illustrating how crossover placement modulates the global shape of the structure.

These results suggest that it is indeed possible to mitigate curvature through careful crossover design. However, in the context of CDL, the observed curvature appears to be a feature rather than a flaw — it is likely essential for enabling chirality-selective behavior. Along the same lines, switching to a multilayered DNA origami design would increase structural rigidity and thereby reduce flexibility, which could in turn diminish chirality selectivity."

Quantifying the curvature of CDL DNA Origami arms:

To quantify the curvature, we selected 10 nucleotides as shown in the below figure. The curve formed by the magenta points was fit with best-fitted 2D circles.

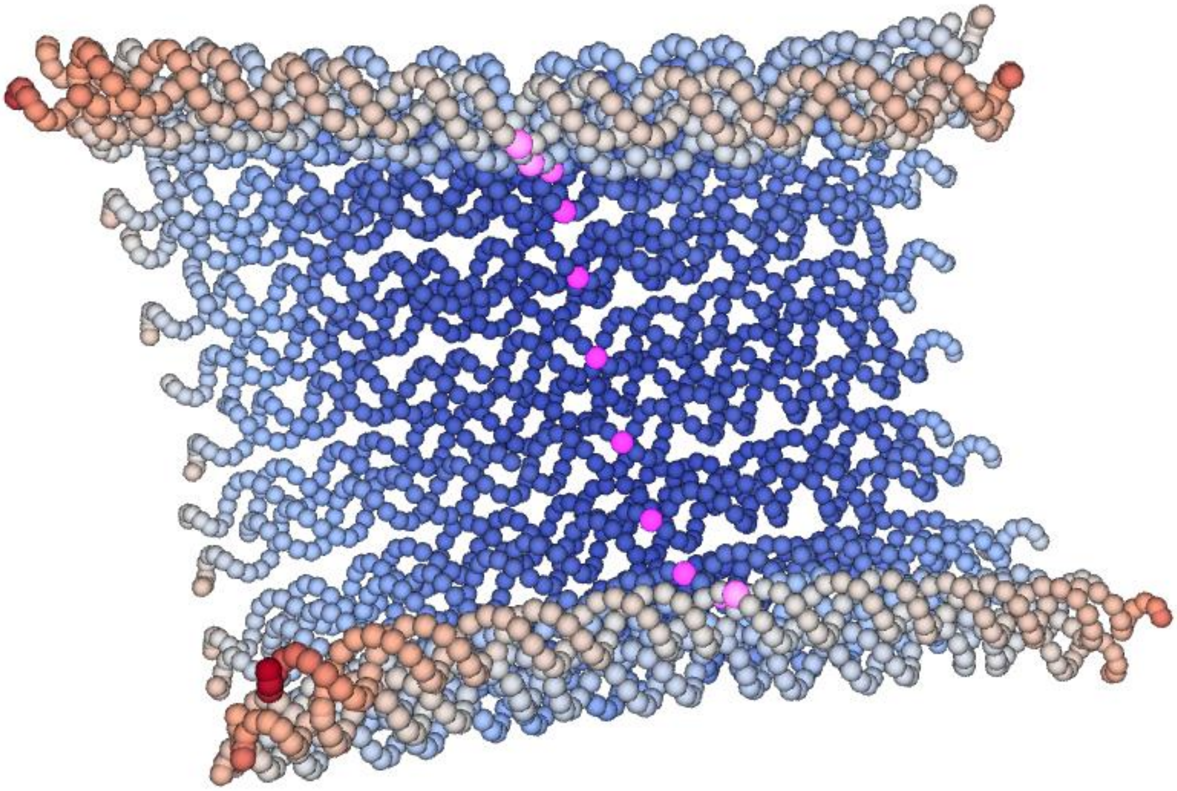

Figure S22. Quantifying the curvature of CDL DNA Origami arm curvature as a function of Na^+ concentration.

Table S19. Corresponding radii and curvatures for the 3 different Na^+ concentrations**.**

**Na concentration (M)**	**curvature (1/nm)**	**bend radius (nm)**
0.5	0.138	7.25
2	0.119	8.40
5	0.111	9.0

References:

[1] B. Shen, V. Linko, K. Tapio, S. Pikker, T. Lemma, A. Gopinath, K. V. Gothelf, M. A. Kostiainen, J. J. Toppari, Plasmonic nanostructures through DNA-assisted lithography. *Sci. Adv.* **2018**, *4*, eaap8978.

[2] A. Suma, E. Poppleton, M. Matthies, P. Šulc, F. Romano, A. A. Louis, J. P. K. Doye, C. Micheletti, L. Rovigatti, TacoxDNA: A user-friendly web server for simulations of complex DNA structures, from single strands to origami. *J. Comput. Chem.* **2019**, *40*, 2586-2595.

[3] E. Poppleton, J. Bohlin, M. Matthies, S. Sharma, F. Zhang, P. Šulc, Design, optimization and analysis of large DNA and RNA nanostructures through interactive visualization, editing and molecular simulation. *Nucleic Acids Res.* **2020**, *48*, e72-e72.

[4] J. Bohlin, M. Matthies, E. Poppleton, J. Procyk, A. Mallya, H. Yan, P. Šulc, Design and simulation of DNA, RNA and hybrid protein–nucleic acid nanostructures with oxView. *Nature Protocols* **2022**, *17*, 1762-1788.
